# netDx: Software for building interpretable patient classifiers by multi-'omic data integration using patient similarity networks

**DOI:** 10.12688/f1000research.26429.2

**Published:** 2021-01-22

**Authors:** Shraddha Pai, Philipp Weber, Ruth Isserlin, Hussam Kaka, Shirley Hui, Muhammad Ahmad Shah, Luca Giudice, Rosalba Giugno, Anne Krogh Nøhr, Jan Baumbach, Gary D. Bader

**Affiliations:** 1 The Donnelly Centre, University of Toronto, Toronto, Canada; 2 Department of Mathematics and Computer Science, University of Southern Denmark, Odense, Denmark; 3 Department of Computer Science, University of Verona, Verona, Italy; 4 The Bioinformatics Centre, Department of Biology, University of Copenhagen, Copenhagen N, Denmark; 5 H. Lundbeck A/S, Copenhagen, Denmark; 6 TUM School of Life Sciences Wiehenstephan, Technical University of Munich, Munich, Germany; 7 Department of Molecular Genetics, University of Toronto, Toronto, Canada; 8 Department of Computer Science, University of Toronto, Toronto, Canada; 9 The Lunenfeld-Tanenbaum Research Institute, Mount Sinal Hospital, Toronto, Canada

**Keywords:** precision medicine, networks, classification, supervised learning, genomics, data integration

## Abstract

Patient classification based on clinical and genomic data will further the goal of precision medicine. Interpretability is of particular relevance for models based on genomic data, where sample sizes are relatively small (in the hundreds), increasing overfitting risk netDx is a machine learning method to integrate multi-modal patient data and build a patient classifier. Patient data are converted into networks of patient similarity, which is intuitive to clinicians who also use patient similarity for medical diagnosis. Features passing selection are integrated, and new patients are assigned to the class with the greatest profile similarity. netDx has excellent performance, outperforming most machine-learning methods in binary cancer survival prediction. It handles missing data – a common problem in real-world data – without requiring imputation. netDx also has excellent interpretability, with native support to group genes into pathways for mechanistic insight into predictive features.

The netDx Bioconductor package provides multiple workflows for users to build custom patient classifiers. It provides turnkey functions for one-step predictor generation from multi-modal data, including feature selection over multiple train/test data splits. Workflows offer versatility with custom feature design, choice of similarity metric; speed is improved by parallel execution. Built-in functions and examples allow users to compute model performance metrics such as AUROC, AUPR, and accuracy. netDx uses RCy3 to visualize top-scoring pathways and the final integrated patient network in Cytoscape. Advanced users can build more complex predictor designs with functional building blocks used in the default design. Finally, the netDx Bioconductor package provides a novel workflow for pathway-based patient classification from sparse genetic data.

## Introduction

**Figure 1.**
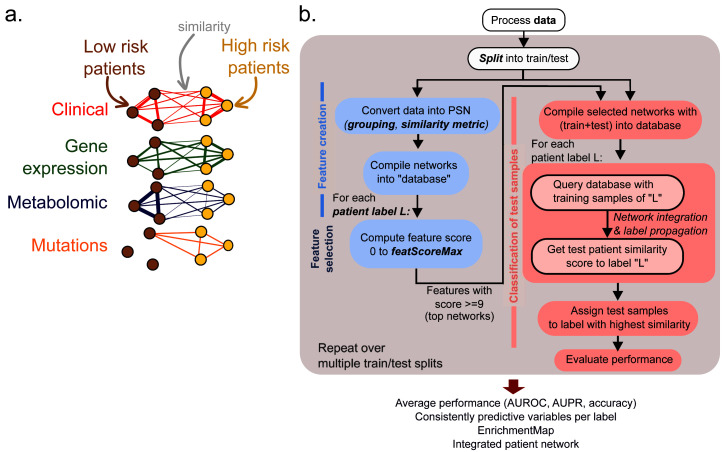
netDx concepts and software workflow. ( **a**) Conceptual visualization of patient similarity networks. Nodes are patients and edge weights measure pairwise similarity. The example shows a two-class problem (high and low risk patients), with four features shown as patient similarity networks: similarity for clinical (red), gene expression (green), metabolomic (blue) and mutation (orange) data. ( **b**) Conceptual workflow for netDx predictor. Samples are split into train and test samples, and training samples are subjected to feature selection (blue flow). Feature selection uses regularized regression to integrate networks, such that networks with non-zero regression weights have their feature score increased. This process is repeated with different subsamples of training data, for a user-provided maximum of times ( *featScoreMax*). This process is repeated for each patient label. Features passing a user-specified threshold are used to classify held-out samples. Test patients are classified by highest similarity. Patient networks that combine training and test patients are then integrated; only networks from features passing selection are used for this step. Label propagation is used to compute similarity of each test patient to training samples from each label; a given patient is assigned to the class with highest similarity. Average model performance is computed by running this whole process over several train/test splits. Features with consistent high scores can be used to classify an independent validation set.

Supervised learning methods are useful in clinical genomics for disease diagnosis, risk stratification for prognosis, and evaluating treatment response. Machine learning is a powerful analytic approach that can identify patterns separating patient groups, but interpreting models remains an active area of research ^1^. Interpretability is desirable to better understand biological mechanism underlying the phenotype and for rational treatment design. It is also important for genomic applications, where most contemporary datasets have fewer than a thousand samples, increasing the risk of overfit models that do not independently replicate. Separately, most machine learning methods do not handle missing data – a common feature of real-world datasets – without prior data imputation or filtering. netDx is a supervised learning algorithm that classifies patients by integrating multimodal patient data ^2^. It is notable among machine learning methods for handling missing data without imputation, and excels at interpretability by enabling users to create biologically-meaningful grouping of features, such as grouping genes into pathway-level features. netDx integrates multi-modal data by converting each layer into a patient similarity network and then integrating these networks ( Figure 1a).

This paper provides an introduction to the R-based software implementation of netDx for the Bioconductor system ^3^ and showcases common use cases. The details of the netDx algorithm and performance have been previously published ^2^, though we provide a brief conceptual summary here ( Figure 1b). As input, the user provides multiple sets of data measured on the same set of labelled patients. The user additionally provides functions to compute pairwise patient similarity and optionally, rules to group measures from each data type into features (feature design). For example, gene expression measures could be grouped into features representing known biological pathways. As with other machine learning methods, the user specifies parameters for training the model, such as the threshold scores for feature selection. Consider an application to predict good or poor patient survival, using tumour-derived gene expression, DNA methylation and proteomic data. In this scenario, netDx is provided with three data tables, one per ‘omic data type, a table with patient identifiers and known labels, and the grouping rule that one feature is to be created per data layer. Patients are then automatically split into training and test samples and feature selection is performed using training samples. netDx uses the given feature processing rules to convert data from different modalities into a common space of patient similarity networks ^1, 2^. Feature selection is performed once per patient label, and features passing selection are used to classify patients from the held-out test data. Performance robustness is evaluated by repeating this feature selection and classification exercise for multiple train/test splits. The final model is created from features that scored highly in feature selection, a step that uses only training samples. A feature may comprise an entire data layer, a single variable, or specified groupings; one example of the last is grouping gene-level measures into pathways, so that each pathway is a separate feature. Interpretability is aided by the pathway-level predictor design, which identifies cellular processes with predictive value.

## Methods

### Implementation

**Figure 2.**
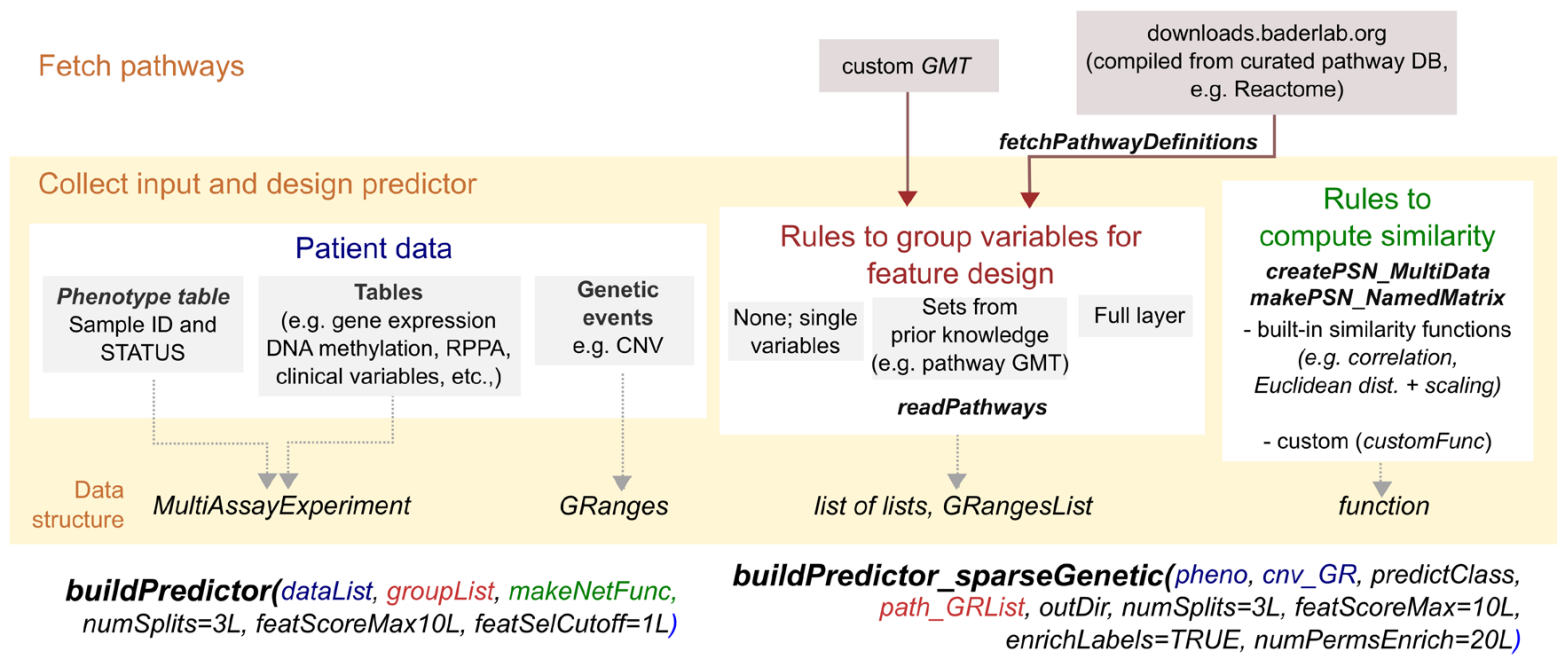
netDx software workflow. The yellow box shows data provided to netDx to build the predictor. See use cases for examples. Patient data is provided as a *MultiAssayExperiment* object, with the patient metadata in the *colData* slot. Variable grouping rules, used for feature design, are provided as a list of lists. The outer list corresponds to a given assay; each entry in the corresponding list corresponds to one group of measures and is used to create a single feature. For instance, in a pathway-based design, one entry in the outer list would be "gene expression", with each inner list containing genes grouped by pathways. In this scenario, each of these gene groupings generates a single pathway-level feature. Pathway definitions can be automatically fetched using the *fetchPathwayDefinitions()* function, or custom definitions can be provided in the GMT file format. For the workflow using sparse genetic data (see *Use Case 3*), such as CNVs, patient CNVs are provided to netDx as a *GRanges* object. In this instance, pathways are provided in *GRangesList* format.

netDx ^3^ is integrated into the Bioconductor system, a high-quality computational biology software framework for genomic data analysis in the statistical programming language R ^4^. Figure 2 shows the workflow for building a model using the netDx software package; Table 1 describes major function calls. netDx uses Bioconductor data structures and mechanisms for fetching and storing data, and representation of input data.

**Table 1.** Major functions for basic and advanced model-building, and result evaluation. Intermediate functions used to prepare data are not shown, but are illustrated in the use cases. The third column shows outdated names for these functions from the software release that accompanied the paper describing the methods.

Function name (v1.1.4)	Purpose
*buildPredictor()*	Turnkey function to build predictor
*buildPredictor_sparseGenetic()*	Turnkey function to build predictor from sparse mutation data of arbitrary ranges (e.g. copy number variants)
*makePSN_NamedMatrix()*	Create PSN from a single data layer (matrix representation)
*createPSN_MultiData()*	Create PSN from multiple data layers. May include calls to *makePSN_NamedMatrix()*
*plotPerf()*	Plot ROC and PR curves, compute AUROC and AUPR
*plotEMap()*	Plot enrichment map in Cytoscape, annotating main themes. Used in pathway-based feature design
*plotIntegratedPatientNetwork()*	Plot patient network by integrating predictive features for all patient labels
*splitTrainTest()*	Randomly split patients into training and test samples
*setupFeatureDB()*	Collects all created features into a database in preparation for feature selection
*compileFeatures()*	Feature selection process. Iterative scoring of input networks based on network integration and regularized regression. Each scoring step is called a “query”. A pre-specified number of queries is run with different subsamples of the training set
*runFeatureSelection()*	Feature selection: Run network integration and label propagation. Used in feature selection for unit increase in network weights. Also used for classification of test samples, where it returns similarity scores
*writeQueryFile()* *runQuery()*	Feature selection: Prepare input for unit-level network integration. Feature selection: Run unit-level network integration. Each run of this step allows unit-level increase of feature scores
*compileFeatureScores()*	Feature selection: Collect feature scores for unit-level network integration and feature scoring steps
*getPatientRankings()*	Model evaluation: Process test patient rankings from label propagation, to compute model performance measures
*predictPatientLabels()*	Model evaluation: Given patient rankings for each label, assign predicted class label to test patients

### Operation

Running netDx requires a machine with one or more cores running Intel processors (1.7 GHz i7 or later) or equivalent, a minimum of 1Gb RAM per thread, and 1Gb disk space. Feature selection is an embarrassingly parallel problem, and we highly recommend running the software on a multi-core machine to reduce compute time. netDx is currently supported on machines running OS X or Unix-based operating systems. The software requires the Java executable (v1.8 or higher) to be available on the system path, and will not work on recent Windows-based operating systems that lack this type of installation. Windows users can access netDx via a Docker image provided at https://hub.docker.com/repository/docker/shraddhapai/netdx. netDx v1.1.4 requires R>=3.6 and BioConductor release 3.11 or higher. When building a predictor, patient data is provided to netDx as a *MultiAssayExperiment* object, a Bioconductor data structure used to represent multi-’omics experiments associated with a given set of samples. In our usage, data types are collected in the *assays* slot of the object; the sole exception is clinical data, which is provided as part of the sample metadata, using the *colData* slot. Grouping rules are provided as a nested *list* object - or list-of-lists ( *groupList*). The outer list consists of one entry per data type, with corresponding groupings in the inner list. Assays names must be identical in the *assays* slot and in *groupList*.

The easiest way to build classifiers is to use the wrapper function, *buildPredictor()*. This function runs feature selection and classification over a specified number of train/test splits, and returns all associated feature scores and detailed classification results in a *list* object. Advanced users can create custom predictor designs by combining the individual steps used in *buildPredictor()* ( Table 1).

## Use cases

This section describes four use cases for building predictors with netDx. The first uses pathway-level features based on gene expression data to generate a binary classifier of breast cancer subtype. The second performs three-way classification of breast cancer subtype by integrating gene expression, DNA methylation and proteomic assays. The third builds a binary classifier of autism spectrum disorder diagnosis from sparse genetic mutations. The fourth involves prediction of tumour stage from somatic mutations that have been desparsified using prior knowledge of gene interaction networks.

### Use case 1: Binary classifier from clinical and transcriptomic data, using pathway-level features

***Introduction.*** In this example, we will build a binary breast tumour Luminal A subtype classifier from clinical data and gene expression data. We will use different rules to create features for each assay. Specifically:

- Clinical measures (e.g. age, stage): Features are defined at the level of *variables*; similarity is defined as normalized difference.
- Gene expression: Features are defined at the level of *pathways*; similarity is defined by pairwise Pearson correlation.

Feature scoring is automatically performed over multiple random splits of the data into train and blind test partitions. Feature selected networks are those that consistently score highly across the multiple splits (e.g. those that score 9 out of 10 in ≥70% of splits).

Conceptually, this is what the higher-level logic looks like for building a predictor over multiple random splits of samples into training and test groups. In the example below, the predictor runs for 100 train/test splits. Within a split, features are scored from 0 to 10. Features scoring ≥9 are used to predict labels on the held-out test set (20%). The example shows pseudocode, not actual netDx function calls:


featScoreMax <- 10   # max. score for a feature in feature selection
featSelCutoff <- 9   # features scoring at least this much are used to 
                       # classify test patients
numSplits <- 100     # number of random train/test splits to run feature 
                       # selection for. Model performance is averaged over
		       # these iterations.

netScores <- list()  # scores from feature selection, one entry per split
perf <- list()       # model performance for each split

for k in 1:numSplits
 [train, test] <- splitData(80:20) # split data using RNG seed
  featScores[[k]] <- runFeatureSelection(train, featScoreMax)
  topFeat[[k]] <- applyFeatCutoff(featScores[[k]])
 perf[[k]] <- evalModelPerf(topFeat[[k]], test)
end


***Setup***


suppressWarnings(suppressMessages(require(netDx)))


***Data.*** In this example, we use curated data from The Cancer Genome Atlas, through the Bioconductor curatedTCGAData package. The goal is to classify a breast tumour into either a Luminal A subtype or otherwise. The predictor integrates clinical variables selected by the user, along with gene expression data.

Here we load the required packages and download clinical and gene expression data.


suppressWarnings(suppressMessages(library(curatedTCGAData)))


List the available data without downloading any:


curatedTCGAData(diseaseCode="BRCA", assays="*",dry.run=TRUE)

##                                         Title DispatchClass
## 31                       BRCA_CNASeq-20160128           Rda
## 32                       BRCA_CNASNP-20160128           Rda
## 33                       BRCA_CNVSNP-20160128           Rda
## 35             BRCA_GISTIC_AllByGene-20160128           Rda
## 36                 BRCA_GISTIC_Peaks-20160128           Rda
## 37     BRCA_GISTIC_ThresholdedByGene-20160128           Rda
## 39  BRCA_Methylation_methyl27-20160128_assays        H5File
## 40      BRCA_Methylation_methyl27-20160128_se           Rds
## 41 BRCA_Methylation_methyl450-20160128_assays        H5File
## 42     BRCA_Methylation_methyl450-20160128_se           Rds
## 43                 BRCA_miRNASeqGene-20160128           Rda
## 44                    BRCA_mRNAArray-20160128           Rda
## 45                     BRCA_Mutation-20160128           Rda
## 46              BRCA_RNASeq2GeneNorm-20160128           Rda
## 47                   BRCA_RNASeqGene-20160128           Rda
## 48                    BRCA_RPPAArray-20160128           Rda


We will work only with the gene expression data in this example:


brca <- suppressMessages(curatedTCGAData("BRCA",c("mRNAArray"),FALSE))


This next code block prepares the TCGA data. In practice you would do this once, and save the data before running netDx, but we run it here in full to see an end-to-end example.


staget <- sub("[abcd]","",sub("t","",colData(brca)$pathology_T_stage))
staget <- suppressWarnings(as.integer(staget))
colData(brca)$STAGE <- staget

pam50 <- colData(brca)$PAM50.mRNA
pam50[which(!pam50 %in% "Luminal A")] <- "notLumA"
pam50[which(pam50 %in% "Luminal A")] <- "LumA"
colData(brca)$pam_mod <- pam50

tmp <- colData(brca)$PAM50.mRNA
idx <- union(which(tmp %in% c("Normal-like","Luminal B","HER2-enriched")),
                      which(is.na(staget)))
pID <- colData(brca)$patientID
tokeep <- setdiff(pID, pID[idx])
brca <- brca[,tokeep,]

# remove duplicate assays mapped to the same sample
smp <- sampleMap(brca)
samps <- smp[which(smp$assay=="BRCA_mRNAArray-20160128"),]
notdup <- samps[which(!duplicated(samps$primary)),"colname"]
brca[[1]] <- suppressMessages(brca[[1]][,notdup])

## harmonizing input:
##   removing 44 sampleMap rows with 'colname' not in colnames of experiments


The predictor will look for columns named ID and STATUS columns in the sample metadata table. netDx uses these to get the patient identifiers and labels, respectively.


pID <- colData(brca)$patientID
colData(brca)$ID <- pID
colData(brca)$STATUS <- colData(brca)$pam_mod


***Design custom patient similarity networks (features).*** netDx provides a set of default functions to compute patient similarity, including Pearson correlation, normalized difference, and scaled Euclidean distance. However, users may choose to define a custom function that takes patient data and variable groupings as input, and returns a set of patient similarity networks (PSN) as output. The user can customize what datatypes are used, how they are grouped, and what defines patient similarity for a given datatype.

When running the predictor (next section), the user simply passes this custom function as an input variable; i.e. the makeNetFunc parameter when calling buildPredictor().

****Note:**** While netDx supports flexible experimental design, the user must ensure that the design, i.e. the similarity metric and variable groupings are appropriate for a given application. Domain knowledge is recommended to support good design.

netDx requires that the makeNetFunc function take some generic parameters as input. These include:

- dataList: the patient data, provided as a MultiAssayExperiment object. Refer to online tutorials for MultiAssayExperiment to see how to construct those objects from data.
- groupList: sets of input data that will define individual networks (e.g. genes grouped into pathways)
- netDir: the directory where the resulting patient similarity networks will be stored.

**dataList**

In this example, the breast cancer data is already provided to us as a MultiAssayExperiment object:


summary(brca)

## Length	      Class             Mode							
## 1 		MultiAssayExperiment 	S4


**groupList**

This object tells the predictor how to group units when constructing a network. For example, genes may be grouped into a patient similarity network representing a pathway. This object is a list; the names match those of dataList while each value is itself a list and reflects a potential network.


groupList <- list()

# genes in mRNA data are grouped by pathways
pathList <- readPathways(fetchPathwayDefinitions("January",2018))

## ---------------------------------------

## Fetching http://download.baderlab.org/EM_Genesets/January_01_2018/Human/symbol/Human_AllPathways_January_01_2018_symbol.gmt

## File: 182107f6006ac_Human_AllPathways_January_01_2018_symbol.gmt

## Read 3028 pathways in total, internal list has 3009 entries

##  FILTER: sets with num genes in [10, 200]

##    => 971 pathways excluded
##    => 2038 left

groupList[["BRCA_mRNAArray-20160128"]] <- pathList[1:3]
# clinical data is not grouped; each variable is its own feature
groupList[["clinical"]] <- list(
       age="patient.age_at_initial_pathologic_diagnosis",
       stage="STAGE"
)



So the groupList variable has one entry per data layer:


summary(groupList)

##                         Length Class  Mode
## BRCA_mRNAArray-20160128 3      -none- list
## clinical                2      -none- list


Each entry contains a list, with one entry per feature. Here we have three pathway-level features for mRNA and two variable-level features for clinical data.

For example, here are the networks to be created with RNA data. Genes corresponding to pathways are to be grouped into individual network. Such a groupList would create pathway-level networks:


groupList[["BRCA_mRNAArray-20160128"]][1:3]

## $UREA_CYCLE
##  [1] "SLC25A15" "CPS1"     "ASL"      "ARG2"     "SLC25A2"  "OTC"     
##  [7] "NMRAL1"   "NAGS"     "ASS1"     "ARG1"    
## 
## $`CDP-DIACYLGLYCEROL_BIOSYNTHESIS_I`
##  [1] "AGPAT1" "GPD2"   "ABHD5"  "GPAT2"  "CDS1"   "LPCAT3" "LPCAT4"
##  [8] "CDS2"   "AGPAT6" "AGPAT5" "MBOAT7" "AGPAT9" "LCLAT1" "MBOAT2"
## [15] "AGPAT4" "GPAM"   "AGPAT3" "AGPAT2"
## 
## $`SUPERPATHWAY_OF_D-_I_MYO__I_-INOSITOL__1,4,5_-TRISPHOSPHATE_METABOLISM`
##  [1] "IPMK"   "INPP5B" "INPP5F" "INPP5D" "MINPP1" "INPP5A" "ITPKA" 
##  [8] "OCRL"   "ITPKC"  "ITPKB"  "SYNJ2"  "INPP5J" "INPP5K" "PTEN"  
## [15] "IMPA2"  "INPP1"  "SYNJ1"  "INPPL1" "IMPA1"  "IMPAD1"


For clinical data, we will define each variable as its own network:


head(groupList[["clinical"]])

## $age
## [1] "patient.age_at_initial_pathologic_diagnosis"
## 
## $stage
## [1] "STAGE"


***Define patient similarity measure for each network.*** This function is defined by the user and tells the predictor how to create networks from the provided input data.

This function requires dataList, groupList, and netDir as input variables. The residual ... parameter is to pass additional variables to makePSN_NamedMatrix(), notably numCores (number of parallel jobs).

In this example, the custom similarity function does the following:

1. Creates *pathway-level networks from RNA* data using the default Pearson correlation measure makePSN_NamedMatrix(writeProfiles=TRUE, ...)

2. Creates *variable-level networks from clinical* data using a custom similarity function of normalized difference: makePSN_NamedMatrix(writeProfiles=FALSE, simMetric="custom", customFunc=normDiff).


makeNets <- function(dataList, groupList, netDir,...) {
     netList <- c() # initialize before is.null() check
     # make RNA nets (NOTE: the check for is.null() is important!)
     # (Pearson correlation)
     if (!is.null(groupList[["BRCA_mRNAArray-20160128"]])) { 
     netList <- makePSN_NamedMatrix(dataList[["BRCA_mRNAArray-20160128"]],
                  rownames(dataList[["BRCA_mRNAArray-20160128"]]),
                  groupList[["BRCA_mRNAArray-20160128"]],
                  netDir,verbose=FALSE, 
                  writeProfiles=TRUE,...) 
     }
    
     # make clinical nets (normalized difference)
     netList2 <- c()
     if (!is.null(groupList[["clinical"]])) {
     netList2 <- makePSN_NamedMatrix(dataList$clinical, 
          rownames(dataList$clinical),
          groupList[["clinical"]],netDir,
          simMetric="custom",customFunc=normDiff, # custom function
          writeProfiles=FALSE,
          sparsify=TRUE,verbose=TRUE,...)
     }
     netList <- c(unlist(netList),unlist(netList2))
     return(netList)
}


**Note:** dataList and groupList are generic containers that can contain whatever object the user requires to create a PSN. **The custom function supports flexible feature design**.

***Build predictor.*** Finally, we call the function that runs the netDx predictor. We provide:

• number of train/test splits over which to collect feature scores and average performance (numSplits),

• maximum score for features in one round of feature selection ( featScoreMax)

• threshold to call feature-selected networks for each train/test split ( featSelCutoff); only features scoring this value or higher will be used to classify test patients, and

• the information to create the PSN, including patient data ( dataList), how variables are to be grouped into networks ( groupList) and the custom function to generate features ( makeNetFunc).

Change numCores to match the number of cores available on your machine for parallel processing.

The call below runs two train/test splits. Within each split, it:

• splits data into train/test using the default split of 80:20

• scores networks between 0 to 2 (i.e. featScoreMax=2)

• uses networks that score ≥1 out of 2 ( featSelCutoff) to classify test samples for that split.

These are unrealistically low values set so the example will run fast. In practice a good starting point is featScoreMax=10, featSelCutoff=9 and numSplits=100, but these parameters may need to be tuned to the sample sizes in the dataset and heterogeneity of the samples. Datasets with high levels of heterogeneity or small sample sizes may benefit from increased sampling – i.e. higher numSplits value. Increasing this setting increases the time to train the model but identifies generalizable patterns over a larger set of random subsamples.



set.seed(42) # make results reproducible
outDir <- sprintf("%s/pred_output",tempdir()) # location for intermediate work
# set keepAllData=TRUE to not delete at the end of the predictor run.
# This can be useful for debugging.
out <- suppressMessages(
   buildPredictor(
      dataList=brca,groupList=groupList,
      makeNetFunc=makeNets,outDir=outDir,
      numSplits=2L,featScoreMax=2L,
      featSelCutoff=1L,
      numCores=1L,
      logging="none")
   )



***Examine output***

The results are stored in the list object returned by the buildPredictor() call. This list contains:

● inputNets: all input networks that the model started with.

● Split<i>: a list with results for each train-test split

– predictions: real and predicted labels for test patients
– accuracy: percent accuracy of predictions
– featureScores: feature scores for each label (list with g entries, where g is number of patient labels). Each entry contains the feature selection scores for the corresponding label.
– featureSelected: vector of features that pass feature selection. List of length g, with one entry per label.

summary(out)

##           Length Class  Mode     
## inputNets 10     -none- character
## Split1     4     -none- list     
## Split2     4     -none- list

summary(out$Split1)

##                 Length Class     
## featureScores      2   -none-    
## featureSelected    2   -none-    
## predictions     2692   data.frame
## accuracy           1   -none-    
##                 Mode   
## featureScores   list   
## featureSelected list   
## predictions     list   
## accuracy        numeric


***Reformat results for further analysis***

This code collects different components of model output to examine the results.


numSplits <- 2
st <- unique(colData(brca)$STATUS)
acc <- c()         # accuracy
predList <- list() # prediction tables

featScores <- list() # feature scores per class
for (cur in unique(st)) featScores[[cur]] <- list()

for (k in 1:numSplits) { 
    pred <- out[[sprintf("Split%i",k)]][["predictions"]];
    # predictions table
    tmp <- pred[,c("ID","STATUS","TT_STATUS","PRED_CLASS",
                     sprintf("%s_SCORE",st))]
     predList[[k]] <- tmp 
     # accuracy
     acc <- c(acc, sum(tmp$PRED==tmp$STATUS)/nrow(tmp))
     # feature scores
     for (cur in unique(st)) {
        tmp <- out[[sprintf("Split%i",k)]][["featureScores"]][[cur]]
        colnames(tmp) <- c("PATHWAY_NAME","SCORE")
        featScores[[cur]][[sprintf("Split%i",k)]] <- tmp
     }
}


***Compute model performance***

After compiling the data above, plot accuracy for each train/test split:


print(acc)

## [1] 0.8507463 0.8059701


**Figure 3.**
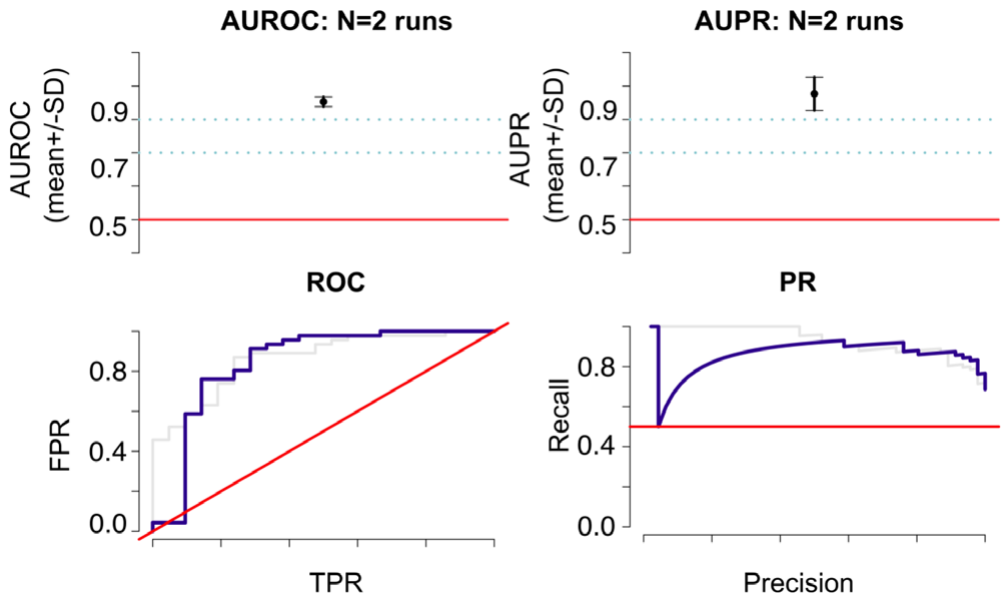
Average model performance for binary classification of breast tumour as "LumA" or "other" using clinical and transcriptomic data. Clockwise from top-left: Mean area under ROC curve across train/test splits; mean area under precision-recall curve; precision-recall curve (average in blue; individual splits in grey); ROC curves (average in blue; individual splits in grey).

Create a ROC curve, a precision-recall curve, and plot average AUROC and AUPR ( Figure 3):


predPerf <- plotPerf(predList, predClasses=st)


***Examine feature scores and consistently high-scoring features.*** Use getNetConsensus() to convert the list data structure into a single table, one per patient label. The rows show train/test splits and the columns show features that consistently perform well.

We then use callFeatSel() to identify features that consistently perform well across the various train/test splits. Because this is a toy example, we set the bar low to get some features. Here we accept a feature if it scores 1 or higher ( fsCutoff=1) in even one split ( fsPctPass=0.05), setting the latter to a low positive fraction.


featScores2 <- lapply(featScores, getNetConsensus)
summary(featScores2)

##         Length Class      Mode
## LumA    3      data.frame list
## notLumA 3      data.frame list

head(featScores2[["LumA"]])

##                                                                     PATHWAY_NAME
## 1                                      CDP-DIACYLGLYCEROL_BIOSYNTHESIS_I.profile
## 2 SUPERPATHWAY_OF_D-_I_MYO__I_-INOSITOL__1,4,5_-TRISPHOSPHATE_METABOLISM.profile
## 3                                                             UREA_CYCLE.profile
## 4                                                                   age_cont.txt
## 5                                                                 stage_cont.txt
##   Split1 Split2
## 1      2      2
## 2      2      2
## 3      2      2
## 4     NA      1
## 5     NA      1


Where features are scored out of 10, a reasonable setting is fsCutoff=9 and fsPctPass=0.7. This setting gives us features that score a minimum of 9 in at least 70% of the train/test splits.


featSelNet <- lapply(featScores2, function(x) {
    callFeatSel(x, fsCutoff=1, fsPctPass=0)
})
print(head(featScores2[["LumA"]]))

##                                                                     PATHWAY_NAME
## 1                                      CDP-DIACYLGLYCEROL_BIOSYNTHESIS_I.profile
## 2 SUPERPATHWAY_OF_D-_I_MYO__I_-INOSITOL__1,4,5_-TRISPHOSPHATE_METABOLISM.profile
## 3                                                             UREA_CYCLE.profile
## 4                                                                   age_cont.txt
## 5                                                                 stage_cont.txt
##   Split1 Split2
## 1      2      2
## 2      2      2
## 3      2      2
## 4     NA      1
## 5     NA      1


***Visualize pathway features as an enrichment map.*** An enrichment map is a network-based visualization of pathway connectivity and is used in netDx to visualize themes in predictive pathway-based features ^5^. It is used in conjunction with the AutoAnnotate Cytoscape app to identify clusters, and apply auto-generated labels to these ^6^.

Use getEMapInput_many() to create the input that helps generate the enrichment map in Cytoscape.


Emap_res <- getEMapInput_many(featScores2,pathList,
     minScore=1,maxScore=2,pctPass=0,out$inputNets,verbose=FALSE)


Write the results to files that Cytoscape can read in:


gmtFiles <- list()
nodeAttrFiles <- list()

for (g in names(Emap_res)) {
    outFile <- sprintf("%s/%s_nodeAttrs.txt",outDir,g)
    write.table(Emap_res[[g]][["nodeAttrs"]],file=outFile,
        sep="\t",col=TRUE,row=FALSE,quote=FALSE)
    nodeAttrFiles[[g]] <- outFile

    outFile <- sprintf("%s/%s.gmt",outDir,g)
    conn <- suppressWarnings(
          suppressMessages(base::file(outFile,"w")))
    tmp <- Emap_res[[g]][["featureSets"]]
    gmtFiles[[g]] <- outFile

    for (cur in names(tmp)) {
        curr <- sprintf("%s\t%s\t%s", cur,cur,
             paste(tmp[[cur]],collapse="\t"))
        writeLines(curr,con=conn)
    }
close(conn)
}


**Figure 4.**
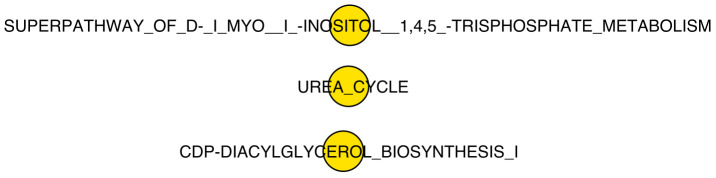
Enrichment map of top-scoring pathway features in tumour classifier example. The small number of nodes reflects the limited number of pathways provided to the toy example model, and also reduced parameter values for model building. See Figure 5 for an example of a more informative enrichment map produced by running a real-world example.

Finally, plot the enrichment map. This step requires Cytoscape to be installed, along with the EnrichmentMap and AutoAnnotate apps. It also requires the Cytoscape application to be open and running on the machine running the code. This block is commented out for automatic builds on Bioconductor, but a screenshot of the intended result is shown below ( Figure 4).


plotEmap(gmtFiles[[1]], nodeAttrFiles[[1]], 
groupClusters=TRUE,hideNodeLabels=TRUE)


This example enrichment map isn’t terribly exciting because of the low number of pathway features permitted, the upper bound on feature selection scores and low number of train/test splits in the demonstration example.

**Figure 5.**
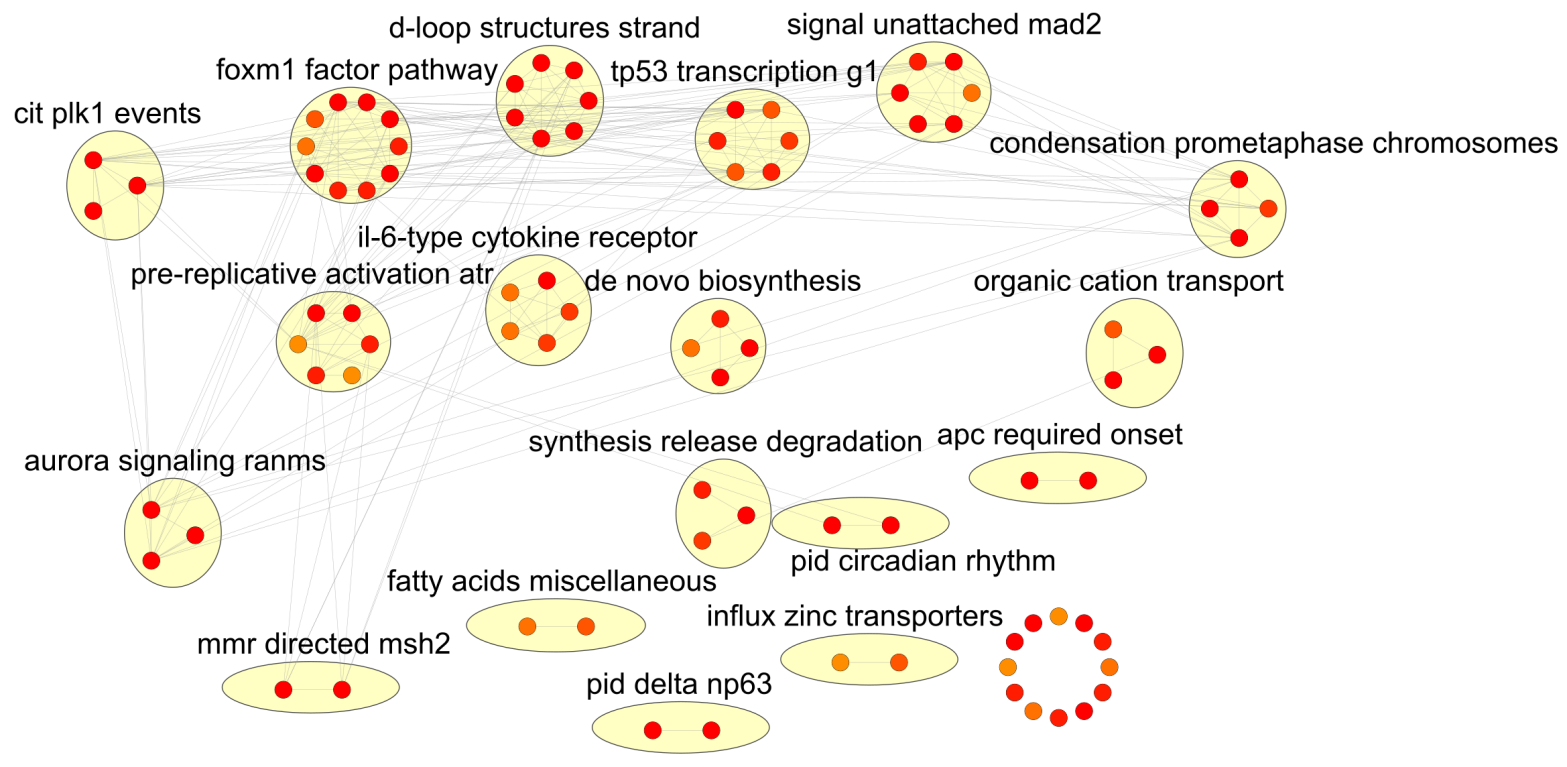
Enrichment map shows consistently high-scoring pathway features when running the breast tumour binary classifier with real-world parameters. This network is generated by running the *plotEmap()* function, which uses the *RCy3* Bioconductor package to programmatically call Cytoscape network visualization software from within R, to run the EnrichmentMap app ^5– 7^. Nodes show pathways features that scored a minimum of 9 out of 10 in feature selection, in at least 70% of train/test splits; node fill indicates feature score. Edges connect pathways with shared genes. The larger yellow bubbles are auto-generated by the AutoAnnotate Cytoscape app ^6, 8^; these thematically group top pathways, by clustering and word-frequency based cluster annotation.

Here is an example of an enrichment map generated by running the above predictor with more real-world parameter values, and all available pathways ( Figure 5):

***Visualize integrated patient similarity network based on top features.*** We apply a threshold to define the most predictive features, and integrate these into a single patient similarity network. Such a network is useful for downstream operations such as ascertaining whether or not classes are significantly separated, and for visualization of results.

Here we define predictive features as those scoring 2 out of 2 in all train/test splits.


featScores2 <- lapply(featScores, getNetConsensus)
featSelNet <- lapply(featScores2, function(x) {
     callFeatSel(x, fsCutoff=2, fsPctPass=1)
})


We next examine the features:


print(featSelNet)

## $LumA
## [1] "CDP-DIACYLGLYCEROL_BIOSYNTHESIS_I.profile"                                     
## [2] "SUPERPATHWAY_OF_D-_I_MYO__I_-INOSITOL__1,4,5_-TRISPHOSPHATE_METABOLISM.profile"
## [3] "UREA_CYCLE.profile"                                                            
## 
## $notLumA
## [1] "SUPERPATHWAY_OF_D-_I_MYO__I_-INOSITOL__1,4,5_-TRISPHOSPHATE_METABOLISM.profile"
## [2] "UREA_CYCLE.profile"                                                            
## [3] "stage_cont.txt"


Create a new groupList limited to top features:


topPath <- gsub(".profile","",
         unique(unlist(featSelNet)))
topPath <- gsub("_cont.txt","",topPath)
# create groupList limited to top features
g2 <- list();
for (nm in names(groupList)) {
    cur <- groupList[[nm]]
    idx <- which(names(cur) %in% topPath)
    message(sprintf("%s: %i pathways", nm, length(idx)))
    if (length(idx)>0) g2[[nm]] <- cur[idx]
}

## BRCA_mRNAArray-20160128: 3 pathways

## clinical: 1 pathways


We plot the integrated patient network based on the features selected above.

In the example below, the networks are integrated by taking the mean of the edge weights ( aggFun="MEAN"). For plotting we retain only the top 5% strongest edges ( topX=0.05).

By setting calcShortestPath=TRUE, the function will also compute the pairwise shortest path for within- and across-group nodes. The result is shown as a set of violin plots and a one-sided Wilcoxon-Mann-Whitney test is used to assign significance.

**Figure 6.**
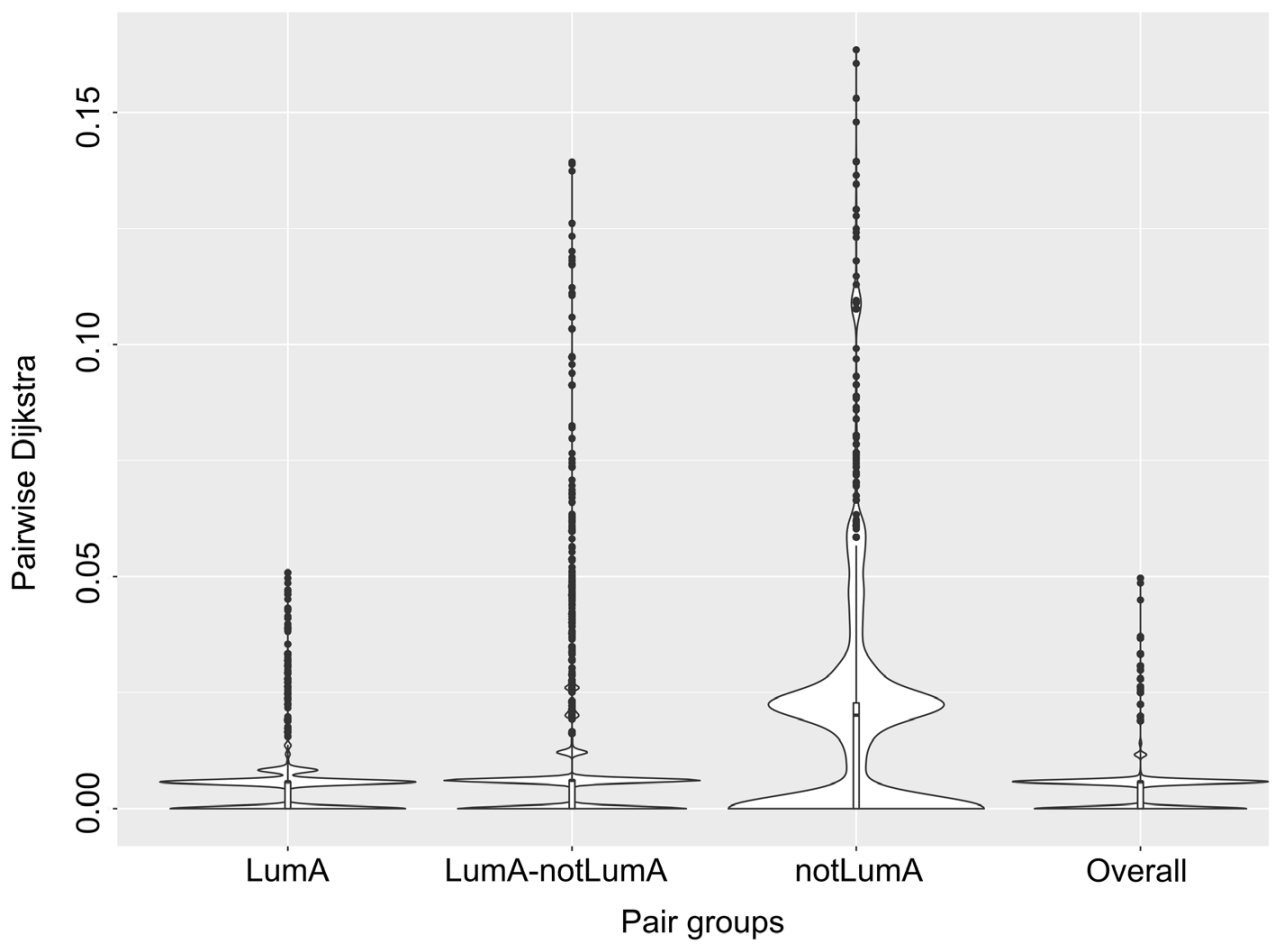
Shortest pairwise patient distances within and among patient classes in the integrated network for breast tumour classification. This visualization and statistic are useful to ascertain whether or not patients of the same label are more similar in the integrated network; having within-class distance be significantly smaller than across-class distance is indicative of good class separation. This graph is generated using the *plotIntegratedPatientNetwork()* function. From left to right, it shows pairwise patient shortest distances: within patients of class "LumA"; between the two class labels; within patients of the residual class "nonLumA"; and between all patients in the network.

**Figure 7.**
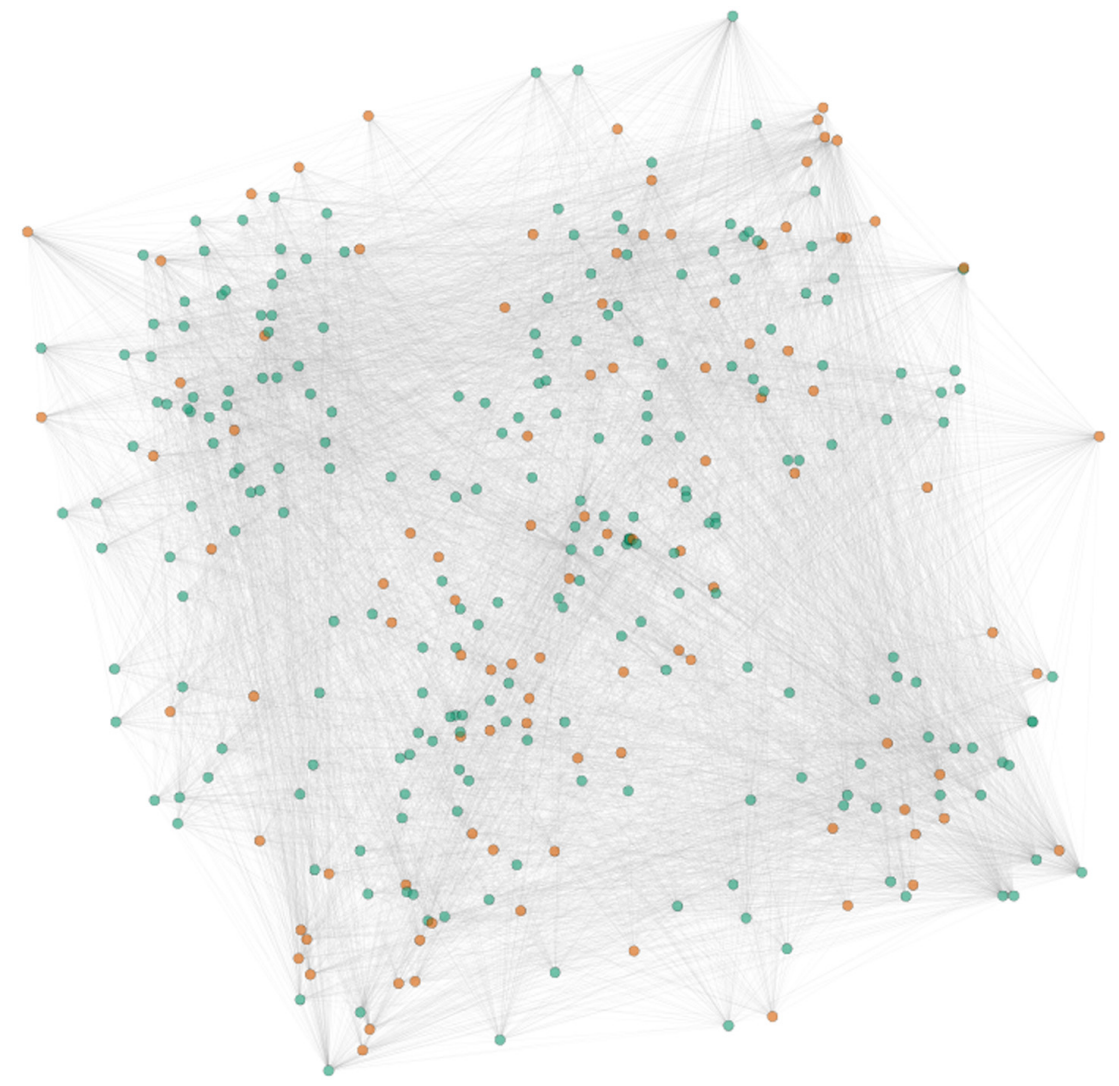
Integrated patient similarity network, generated by combining all networks that consistently pass feature selection. This network is generated by calling *plotIntegratedPatientNetwork()* and uses RCy3 to programmatically generate the network in Cytoscape ^7, 8^. This network uses features that scored 2 out of 2 in all train-test splits. For visualization, only the top 8% most-distant edges are shown. Nodes are patients, and edges weights show average similarity across all features passing feature selection. Node fills indicate patient label, with “LumA” in green and “nonLumA” in orange.

As with plotEMap(), this method must be run on a computer with Cytoscape installed and running. To bypass plotting the PSN in Cytoscape, set plotCytoscape to FALSE. This function call computes shortest-path distances within- and among clusters ( Figure 6) and plots the integrated PSN ( Figure 7). The resulting network is shown below ( Figure 7).


psn <- suppressMessages(
   plotIntegratedPatientNetwork(
       brca,
       groupList=g2, 
       makeNetFunc=makeNets,
       aggFun="MEAN",topX=0.08,
       numCores=1L,calcShortestPath=TRUE,
       showStats=FALSE,
       verbose=FALSE, plotCytoscape=FALSE)
)


**Figure 8.**
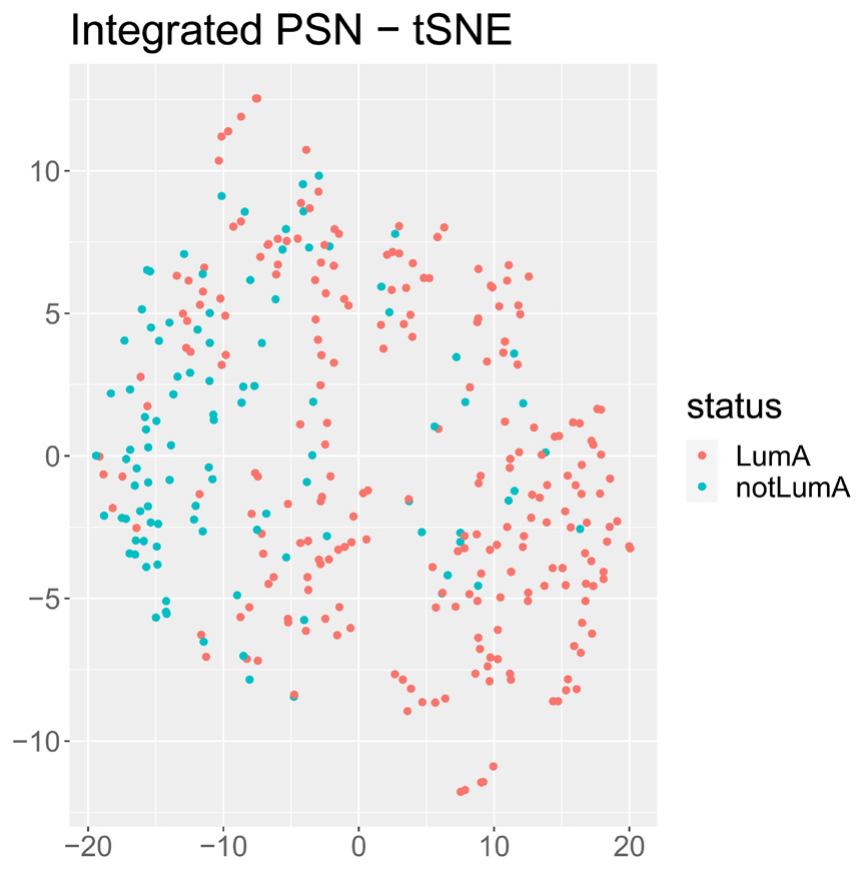
tSNE plot of integrated patient similarity network based on features passing selection.


The integrated PSN can also be visualized as a tSNE plot ( Figure 8).

tsne <- plot_tSNE(psn$patientSimNetwork_unpruned,colData(brca))



summary(tsne)

##                     Length Class  Mode   
## N                     1    -none- numeric
## Y                   662    -none- numeric
## costs               331    -none- numeric
## itercosts            20    -none- numeric
## origD                 1    -none- numeric
## perplexity            1    -none- numeric
## theta                 1    -none- numeric
## max_iter              1    -none- numeric
## stop_lying_iter       1    -none- numeric
## mom_switch_iter       1    -none- numeric
## momentum              1    -none- numeric
## final_momentum        1    -none- numeric
## eta                   1    -none- numeric
## exaggeration_factor   1    -none- numeric


### Use case 2: Three-way classifier with clinical and three types of 'omic data

***Introduction.*** In this example, we will use clinical data and three types of ’omic data - gene expression, DNA methylation and proteomic data - to classify breast tumours as being one of three types: Luminal A, Luminal B, or Basal. This example is an extension of the one used to build a binary classifier (see *Use Case 1*).

We also use several strategies and definitions of similarity to create features:

• Clinical variables: Each *variable* (e.g. age) is its own feature; similarity is defined as *normalized difference*.

• Gene expression: Features are defined at the level of *pathways*; i.e. a feature groups genes within the pathway. Similarity is defined as pairwise *Pearson correlation.*

• Proteomic and methylation data: Features are defined at the level of the entire *data layer*; a single feature is created for all of proteomic data, and the same for methylation. Similarity is defined as pairwise *Pearson correlation*.

***Setup.*** Load the netDx package.


suppressWarnings(suppressMessages(require(netDx)))


***Data.*** For this example, we download data from The Cancer Genome Atlas through the Bioconductor curatedTCGAData package. The fetch command automatically creates a MultiAssayExperiment object containing the data.


suppressMessages(library(curatedTCGAData))


We use the curatedTCGAData() command to explore available data types in the breast cancer dataset.


curatedTCGAData(diseaseCode="BRCA", assays="*",dry.run=TRUE)

##                                         Title DispatchClass
## 31                       BRCA_CNASeq-20160128           Rda
## 32                       BRCA_CNASNP-20160128           Rda
## 33                       BRCA_CNVSNP-20160128           Rda
## 35             BRCA_GISTIC_AllByGene-20160128           Rda
## 36                 BRCA_GISTIC_Peaks-20160128           Rda
## 37     BRCA_GISTIC_ThresholdedByGene-20160128           Rda
## 39  BRCA_Methylation_methyl27-20160128_assays        H5File
## 40      BRCA_Methylation_methyl27-20160128_se           Rds
## 41 BRCA_Methylation_methyl450-20160128_assays        H5File
## 42     BRCA_Methylation_methyl450-20160128_se           Rds
## 43                 BRCA_miRNASeqGene-20160128           Rda
## 44                    BRCA_mRNAArray-20160128           Rda
## 45                     BRCA_Mutation-20160128           Rda
## 46              BRCA_RNASeq2GeneNorm-20160128           Rda
## 47                   BRCA_RNASeqGene-20160128           Rda
## 48                    BRCA_RPPAArray-20160128           Rda


In this call we fetch only the gene expression, proteomic and methylation data; setting dry.run=FALSE initiates the fetching of the data.


brca <- suppressWarnings(suppressMessages(
   curatedTCGAData("BRCA",
                 c("mRNAArray","RPPA*","Methylation_methyl27*"),
    dry.run=FALSE)))


This next code block prepares the TCGA data. In practice this is performed once, and the resulting data is saved before running netDx, but we run it here to see an end-to-end example.


# prepare clinical variable - stage
staget <- sub("[abcd]","",sub("t","",colData(brca)$pathology_T_stage))
staget <- suppressWarnings(as.integer(staget))
colData(brca)$STAGE <- staget

# exclude normal, HER2 (small num samples)
pam50 <- colData(brca)$PAM50.mRNA
idx <- union(which(pam50 %in% c("Normal-like","HER2-enriched")), 
    which(is.na(staget)))
idx <- union(idx, which(is.na(pam50)))
pID <- colData(brca)$patientID
tokeep <- setdiff(pID, pID[idx])
brca <- brca[,tokeep,]

pam50 <- colData(brca)$PAM50.mRNA
colData(brca)$pam_mod <- pam50

# remove duplicate names
smp <- sampleMap(brca)
for (nm in names(brca)) {
    samps <- smp[which(smp$assay==nm),]
    notdup <- samps[which(!duplicated(samps$primary)),"colname"]
    brca[[nm]] <- suppressMessages(brca[[nm]][,notdup])
}


The important thing is to create ID and STATUS columns in the sample metadata slot. netDx uses these to get the patient identifiers and labels, respectively.


pID <- colData(brca)$patientID
colData(brca)$ID <- pID
colData(brca)$STATUS <- gsub(" ","_",colData(brca)$pam_mod)


***Rules to create features (patient similarity networks).*** We will group gene expression data by pathways and clinical data by single variables. We will treat methylation and proteomic data each as a single feature, so each of those groups will contain the entire input table for those corresponding data types.

In the code below, we fetch pathway definitions from January 2018 from a source that auto-compiles these from curated pathway databases ( http://download.baderlab.org/EM_Genesets). We choose the January 2018 source to be consistent with earlier published work, but normally the latest source would be downloaded. We group gene expression measures by pathways.

Grouping rules are accordingly created for the clinical, methylation and proteomic data.


groupList <- list()

# genes in mRNA data are grouped by pathways
pathList <- readPathways(fetchPathwayDefinitions("January",2018))

## ---------------------------------------

## Fetching http://download.baderlab.org/EM_Genesets/January_01_2018/Human/symbol/Human_AllPathways_January_01_2018_symbol.gmt

## File: 182107f6006ac_Human_AllPathways_January_01_2018_symbol.gmt

## Read 3028 pathways in total, internal list has 3009 entries

##  FILTER: sets with num genes in [10, 200]

##    => 971 pathways excluded
##    => 2038 left

groupList[["BRCA_mRNAArray-20160128"]] <- pathList[1:3]
# clinical data is not grouped; each variable is its own feature
groupList[["clinical"]] <- list(
       age="patient.age_at_initial_pathologic_diagnosis",
        stage="STAGE"
)
# for methylation generate one feature containing all probes
# same for proteomics data
tmp <- list(rownames(experiments(brca)[[2]]));
names(tmp) <- names(brca)[2]
groupList[[names(brca)[2]]] <- tmp

tmp <- list(rownames(experiments(brca)[[3]]));
names(tmp) <- names(brca)[3]
groupList[[names(brca)[3]]] <- tmp


***Define patient similarity for each network.*** We provide netDx with a custom function to generate similarity networks (i.e. features). The first block tells netDx to generate correlation-based networks using everything but the clinical data. This is achieved by the call:


makePSN_NamedMatrix(..., writeProfiles=TRUE,...)`


To make features from single measures using clinical data, the second block makes a slightly-modified call to makePSN_NamedMatrix(), this time requesting the use of the normalized difference similarity metric. This is achieved by calling:


makePSN_NamedMatrix(,..., 
                       simMetric="custom", customFunc=normDiff,
                       writeProfiles=FALSE)


normDiff is a function provided in the netDx package, but the user may define custom similarity functions in this block of code and pass those to makePSN_NamedMatrix(), using the customFunc parameter.


makeNets <- function(dataList, groupList, netDir,...) {
    netList <- c() # initialize before is.null() check
    # correlation-based similarity for mRNA, RPPA and methylation data
    # (Pearson correlation)
    for (nm in setdiff(names(groupList),"clinical")) {
       # NOTE: the check for is.null() is important!
        if (!is.null(groupList[[nm]])) {
        netList <- makePSN_NamedMatrix(dataList[[nm]],
                       rownames(dataList[[nm]]),
                    groupList[[nm]],netDir,verbose=FALSE,
                      writeProfiles=TRUE,...) 
        }
    }
    
    # make clinical nets (normalized difference)
    netList2 <- c()
    if (!is.null(groupList[["clinical"]])) {
    netList2 <- makePSN_NamedMatrix(dataList$clinical, 
         rownames(dataList$clinical),
         groupList[["clinical"]],netDir,
         simMetric="custom",customFunc=normDiff, # custom function
         writeProfiles=FALSE,
         sparsify=TRUE,verbose=TRUE,...)
     }
     netList <- c(unlist(netList),unlist(netList2))
     return(netList)
}


***Build predictor.*** Finally, we make the call to build the predictor.


set.seed(42) # set a custom seed to make results reproducible

# location for intermediate work
# set keepAllData to TRUE to not delete at the end of the 
# predictor run.
# This can be useful for debugging.
outDir <- paste(tempdir(),"pred_output",sep=getFileSep()) 

numSplits <- 2L
out <- suppressMessages(
   buildPredictor(dataList=brca,groupList=groupList,
      makeNetFunc=makeNets,outDir=outDir,
      numSplits=numSplits, featScoreMax=2L, featSelCutoff=1L,
       numCores=1L)
)

## function(dataList, groupList, netDir,...) {
##  netList <- c() # initialize before is.null() check
##  # correlation-based similarity for mRNA, RPPA and methylation data
##  # (Pearson correlation)
##  for (nm in setdiff(names(groupList),"clinical")) {
##     # NOTE: the check for is.null() is important!
##      if (!is.null(groupList[[nm]])) {
##      netList <- makePSN_NamedMatrix(dataList[[nm]],
##                   rownames(dataList[[nm]]),
##                    groupList[[nm]],netDir,verbose=FALSE,
##                   writeProfiles=TRUE,...) 
##      }
##  }
##  
##  # make clinical nets (normalized difference)
##  netList2 <- c()
##  if (!is.null(groupList[["clinical"]])) {
##  netList2 <- makePSN_NamedMatrix(dataList$clinical, 
##      rownames(dataList$clinical),
##      groupList[["clinical"]],netDir,
##      simMetric="custom",customFunc=normDiff, # custom function
##      writeProfiles=FALSE,
##      sparsify=TRUE,verbose=TRUE,...)
##  }
##  netList <- c(unlist(netList),unlist(netList2))
##  return(netList)
## }
##             IS_TRAIN
## STATUS       TRAIN TEST
##   Basal-like    77   20
##   Luminal_A    184   46
##   Luminal_B    101   26
## 
## Luminal_A   nonpred      <NA> 
##       184       178         0 
## 
## Basal-like    nonpred       <NA> 
##         77        285          0 
## 
## Luminal_B   nonpred      <NA> 
##       101       261         0 
##             IS_TRAIN
## STATUS       TRAIN TEST
##   Basal-like    77   20
##   Luminal_A    184   46
##   Luminal_B    101   26
## 
## Luminal_A   nonpred      <NA> 
##       184       178         0 
## 
## Basal-like    nonpred       <NA> 
##         77        285          0 
## 
## Luminal_B   nonpred      <NA> 
##       101       261         0


Compute accuracy for three-way classification:


# Average accuracy
st <- unique(colData(brca)$STATUS) 
acc <- matrix(NA,ncol=length(st),nrow=numSplits) 
colnames(acc) <- st 
for (k in 1:numSplits) { 
     pred <- out[[sprintf("Split%i",k)]][["predictions"]];
     tmp <- pred[,c("ID","STATUS","TT_STATUS","PRED_CLASS",
                        sprintf("%s_SCORE",st))]
     for (m in 1:length(st)) {
        tmp2 <- subset(tmp, STATUS==st[m])
        acc[k,m] <- sum(tmp2$PRED==tmp2$STATUS)/nrow(tmp2)
     }
}
print(round(acc*100,2))

##      Luminal_A Basal-like Luminal_B
## [1,]     57.14        100     28.57
## [2,]     58.62        100     45.00


On examining the confusion matrix above, we can see that the model perfectly classifies basal tumours, but performs poorly in distinguishing between the two types of luminal tumours. This performance is unsurprising because luminal and basal tumours have different molecular characteristics, with the latter being ER- tumours; in contrast, both Luminal A and B are both types of ER+ tumours ^9^.


res <- out$Split1$predictions
print(table(res[,c("STATUS","PRED_CLASS")]))

##             PRED_CLASS
## STATUS       Basal-like Luminal_A
##   Basal-like         14         0
##   Luminal_A           4        16
##   Luminal_B           4         6
##             PRED_CLASS
## STATUS       Luminal_B
##   Basal-like         0
##   Luminal_A          8
##   Luminal_B          4


### Use case 3: Binary classifier using sparse genetic data and pathway-level features

netDx natively handles missing data, making it suitable to build predictors with sparse genetic data such as somatic DNA mutations, frequently seen in cancer, and from DNA copy number variations (CNVs). netDx handles missing data at two levels. First, netDx uses patient similarity networks, not input data, as its features. Missing data can be handled by the similarity metric used to make this conversion. e.g. If similarity is defined as the Pearson correlation between gene expression measures at the pathway level, then omitting missing genes from the correlation calculation still allows the correlations, and thus the pathway-level network, to be computed. Where patients are missing a particular feature, the network integration step uses what information it has. For example, in a scenario where the data consist of transcriptomic and proteomic measures, if a patient is missing transcriptomic data, the integration step will use only the proteomic data (network edges) for that patient.

**Figure 9.**
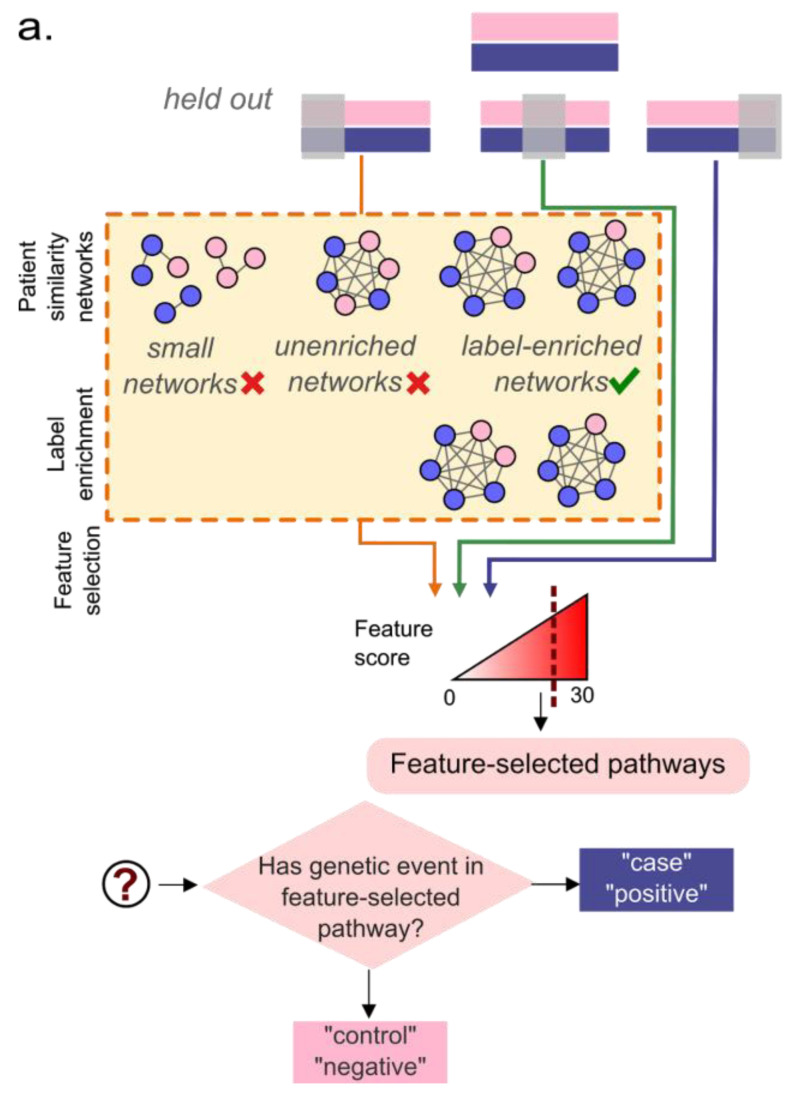
Predictor design for binary classification of case/control diagnosis in netDx, starting from rare CNVs. CNVs are grouped into pathway-level features and patient similarity is binary; i.e. two patients have similarity of one if they share CNVs in genes from the same pathway. Feature selection is iteratively performed on independent thirds of the sample set. This design uses an additional label enrichment step that precedes feature selection. Label enrichment filters out networks with insufficient bias towards case-case edges, using a label-based permutation approach. Networks with significant label enrichment are used in feature selection. Scores from all three feature-selection splits are added to get a final score for each feature, with a maximum attainable score of 30. Test patients are classified as cases if they carry a CNV in a pathway that passes feature selection.

This example demonstrates how to use netDx to build a predictor from sparse genetic data. Here we build a case/control classifier for autism spectrum disorder (ASD) diagnosis, starting from rare CNVs; for this, we use data from Pinto *et al.* ^10^. The design for this predictor is shown in Figure 9.

***Design and adapting the algorithm for sparse event data.*** In this design, we group CNVs by pathways. The logic behind the grouping is prior evidence showing that genetic events in diseases tend to converge on cellular processes of relevance to the pathophysiology of the disease ^10^.

**Binary similarity and label enrichment**

In this design, similarity is defined as a binary function, a strategy that has advantages and drawbacks. In plain terms, ****if two patients share a mutation in a pathway, their similarity for that pathway is 1; otherwise it is zero.**** This binary definition, while conceptually intuitive, increases the false positive rate in the netDx feature selection step. That is, networks with even a single case sample will get a high feature score, regardless of whether that network is enriched for case samples.

To counter this problem, we introduce a ****label-enrichment**** step in the feature selection. A bias measure is first computed for each network, such that a network with only cases scores +1; one with only controls scores -1; and one with an equal number of both has a score of zero. Label-enrichment compares the bias in each real network, to the bias in that network in label-permuted data. It then assigns an empirical p-value for the proportion of times a label-permuted network has a bias as high as the real network. Only networks with a p-value below a user-assigned threshold (default: 0.07) pass label-enrichment, and feature selection is limited to these networks. In netDx, label-enrichment is enabled by setting enrichLabels=TRUE in the call to buildPredictor_sparseGenetic().

**Cumulative feature scoring**

The other difference between this design and those with non-sparse data, is the method of scoring features ( Figure 9). The user specifies a parameter which indicates the number of times to split the data and run feature selection. The algorithm then runs feature selection numSplits times, each time leaving 1/numSplits of the samples out. In each split, features are scored between zero and featScoreMax, using the same approach as is used for continuous-valued input. Feature scores are then added across the splits so that a feature can score as high as numSplits*featScoreMax.

**Evaluating model performance**

For a given cutoff for features, a patient is called a “case” if they have a genetic event in pathways that pass feature selection at that cutoff; otherwise, at that cutoff, they are labelled a “control”. These calls are used to generate the false positive and true positive rates across the various cutoffs, which ultimately generates a ROC curve.

***Setup***


suppressMessages(require(netDx))
suppressMessages(require(GenomicRanges))


***Data.*** CNV coordinates are read in, and converted into a GRanges object. As always, the sample metadata table, here the pheno object, must have ID and STATUS columns.


outDir <- sprintf("%s/200129_threeWay",tempdir())
if (file.exists(outDir)) unlink(outDir,recursive=TRUE); 
dir.create(outDir)

cat("* Setting up sample metadata\n")

## * Setting up sample metadata

phenoFile <- sprintf("%s/extdata/AGP1_CNV.txt",path.package("netDx"))
pheno   <- read.delim(phenoFile,sep="\t",header=TRUE,as.is=TRUE)
colnames(pheno)[1] <- "ID"
head(pheno)

##        ID seqnames    start      end    Gene_symbols Pathogenic STATUS
## 3  1020_4     chr3  4110452  4145874                         no   case
## 4  1030_3    chr10 56265896 56361311                         no   case
## 5  1030_3     chr7 64316996 64593616 ZNF92,LOC441242         no   case
## 7  1045_3     chr3 83206919 83239473                         no   case
## 11 1050_3     chr6 57021412 57062509        KIAA1586         no   case
## 16 1116_4     chr1 30334653 30951250                         no   case

cnv_GR    <- GRanges(pheno$seqnames,IRanges(pheno$start,pheno$end),
                           ID=pheno$ID,LOCUS_NAMES=pheno$Gene_symbols)
pheno <- pheno[!duplicated(pheno$ID),]


***Group CNVs by pathways.*** The fetchPathwayDefinitions() function downloads pathway definitions from baderlab.org but users may provide custom .gmt files as well. We use the BiocFileCache package to download gene definitions for the hg18 genome build, and convert these a GRanges object. The function mapNamedRangesToSets() is used to group this GRanges object into pathway-level sets.


pathFile <- fetchPathwayDefinitions("February",2018,verbose=TRUE)

## Fetching http://download.baderlab.org/EM_Genesets/February_01_2018/Human/symbol/Human_AllPathways_February_01_2018_symbol.gmt

pathwayList <- readPathways(pathFile)

## ---------------------------------------

## File: f72c2f3fae_Human_AllPathways_February_01_2018_symbol.gmt

## Read 3199 pathways in total, internal list has 3163 entries

##  FILTER: sets with num genes in [10, 200]

##    => 1044 pathways excluded

##    => 2119 left

suppress(Messagesrequire(BiocFileCache))
geneURL <- paste("http://download.baderlab.org/netDx/",
    "supporting_data/refGene.hg18.bed",sep="")
cache <- rappdirs::user_cache_dir(appname = "netDx")
bfc <- BiocFileCache::BiocFileCache(cache,ask=FALSE)
geneFile <- bfcrpath(bfc, geneURL)
genes <- read.delim(geneFile,sep="\t",header=FALSE,as.is=TRUE)
genes <- genes[which(genes[,4]!=""),]
gene_GR     <- GRanges(genes[,1],IRanges(genes[,2],genes[,3]),
   name=genes[,4])


Group gene extents into pathway-based sets, which effectively creates grouping rules for netDx. The function mapNamedRangesToSets() does this grouping, generating a GRangesList object.


path_GRList <- mapNamedRangesToSets(gene_GR,pathwayList)


***Run predictor.*** Once the phenotype matrix and grouping rules are set up, the predictor is called using buildPredictor_sparseGenetic(). Note that unlike with non-sparse data, the user does not provide a custom similarity function in this application; currently, the only option available is the binary similarity defined above. As discussed above, setting enrichLabels=TRUE to enable label-enrichment is highly recommended to reduce false positive rate.


predictClass    <- "case"
out <- suppressMessages(
   buildPredictor_sparseGenetic(pheno, cnv_GR, predictClass,
                                 path_GRList,outDir,
                                 numSplits=3L, featScoreMax=3L,
                                 enrichLabels=TRUE,numPermsEnrich=20L,
                                 numCores=2L)
)
##          TT_STATUS
## STATUS    TEST TRAIN
##   case     188   376
##   control  208   418
## [1] 794
##    user  system elapsed 
##   0.681   0.234  11.180 
##    Min. 1st Qu.  Median    Mean 3rd Qu.    Max. 
## -1.0000 -0.7143  0.2000  0.1505  1.0000  1.0000 
## [1] 363
## Time difference of 7.545976 secs
##          TT_STATUS
## STATUS    TEST TRAIN
##   case     188   376
##   control  208   418
## [1] 794
##    user  system elapsed 
##   0.583   0.091   9.431 
##    Min. 1st Qu.  Median    Mean 3rd Qu.    Max. 
## -1.0000 -0.6295  0.1667  0.1269  1.0000  1.0000 
## [1] 392
## Time difference of 12.61768 secs
##          TT_STATUS
## STATUS    TEST TRAIN
##   case     188   376
##   control  210   416
## [1] 792
##    user  system elapsed 
##   0.972   0.146  12.872 
##    Min. 1st Qu.  Median    Mean 3rd Qu.    Max. 
## -1.0000 -0.5668  0.2000  0.1523  1.0000  1.0000 
## [1] 484
## Time difference of 16.2142 secs


**Figure 10.**
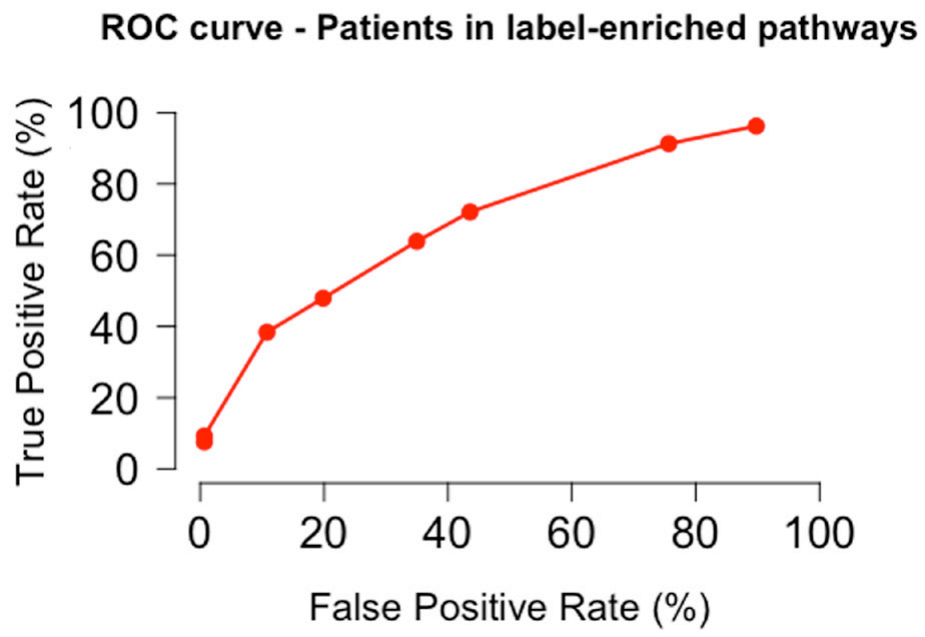
ROC curve for case-control classification in autism, using rare copy number variations (CNV) in pathway genes. In this design, patients are classified as cases if they carry a CNV in pathways passing feature selection, and controls otherwise. Each dot in the graph shows the sensitivity/specificity for a given cutoff for feature selection.


***Plot results.*** Feature selection identifies pathways that are consistently enriched for the label of interest; here, “case” status. From the diagnostic point of view, a patient with a genetic event in a selected feature - here, a CNV in a feature-selected pathway - is labelled a “case”. “True positives” are therefore cases with CNVs in feature-selected pathways, while “false positives” are controls with CNVs in feature-selected pathways. These definitions are used to compute the ROC curve below ( Figure 10).

dat <- out$performance_denEnrichedNets
plot(0,0,type="n",xlim=c(0,100),ylim=c(0,100),
    las=1, xlab="False Positive Rate (%)", 
    ylab="True Positive Rate (%)",
    bty='n',cex.axis=1.5,cex.lab=1.3,
    main="ROC curve - Patients in label-enriched pathways")
points(dat$other_pct,dat$pred_pct,
      col="red",type="o",pch=16,cex=1.3,lwd=2)


We can also compute the AUROC and AUPR. 


tmp <- data.frame(  
    score=dat$score,
    tp=dat$pred_ol,fp=dat$other_ol,
    # tn: "-" that were correctly not called
    tn=dat$other_tot - dat$other_ol,
    # fn: "+" that were not called 
    fn=dat$pred_tot - dat$pred_ol) 

stats <- netDx::perfCalc(tmp)
tmp <- stats$stats
message(sprintf("PRAUC = %1.2f\n", stats$prauc))

## PRAUC = 0.63

message(sprintf("ROCAUC = %1.2f\n", stats$auc))

## ROCAUC = 0.70


This predictor performs outperforms previous CNV-based classifiers ^11^; in a real-world scenario this model would need to be validated on an independent dataset. In our experience, using a combination of sparse genetic data and binary similarity makes classifiers prone to overfitting. Measures commonly used to mitigate overfitting include training the model on larger datasets, and larger number of train/test splits are advised.

Pathway scores are also added across the splits, for a total of 9 across the 3 splits (3 + 3 + 3).


# now get pathway score
tmp <- out$cumulativeFeatScores
rownames(tmp) <- NULL
print(head(tmp))

##                                                      PATHWAY_NAME SCORE
## 1 NEUROTRANSMITTER_RECEPTORS_AND_POSTSYNAPTIC_SIGNAL_TRANSMISSION     8
## 2                                              HUNTINGTON_DISEASE     7
## 3              NICOTINIC_ACETYLCHOLINE_RECEPTOR_SIGNALING_PATHWAY     6
## 4                          BETA-CATENIN_INDEPENDENT_WNT_SIGNALING     6
## 5                                                 G2_M_TRANSITION     6
## 6                                          MITOTIC_G2-G2_M_PHASES     6


As before, running the predictor with all possible pathway-related features and realistic training parameters, such as numPermsEnrich=200L, featScoreMax=10L, numSplits=3L identifies a much richer set of themes related to synaptic transmission and cell proliferation, consistent with the known biology of ASD as well as those identified in the original publication ^10^.

**Figure 11.**
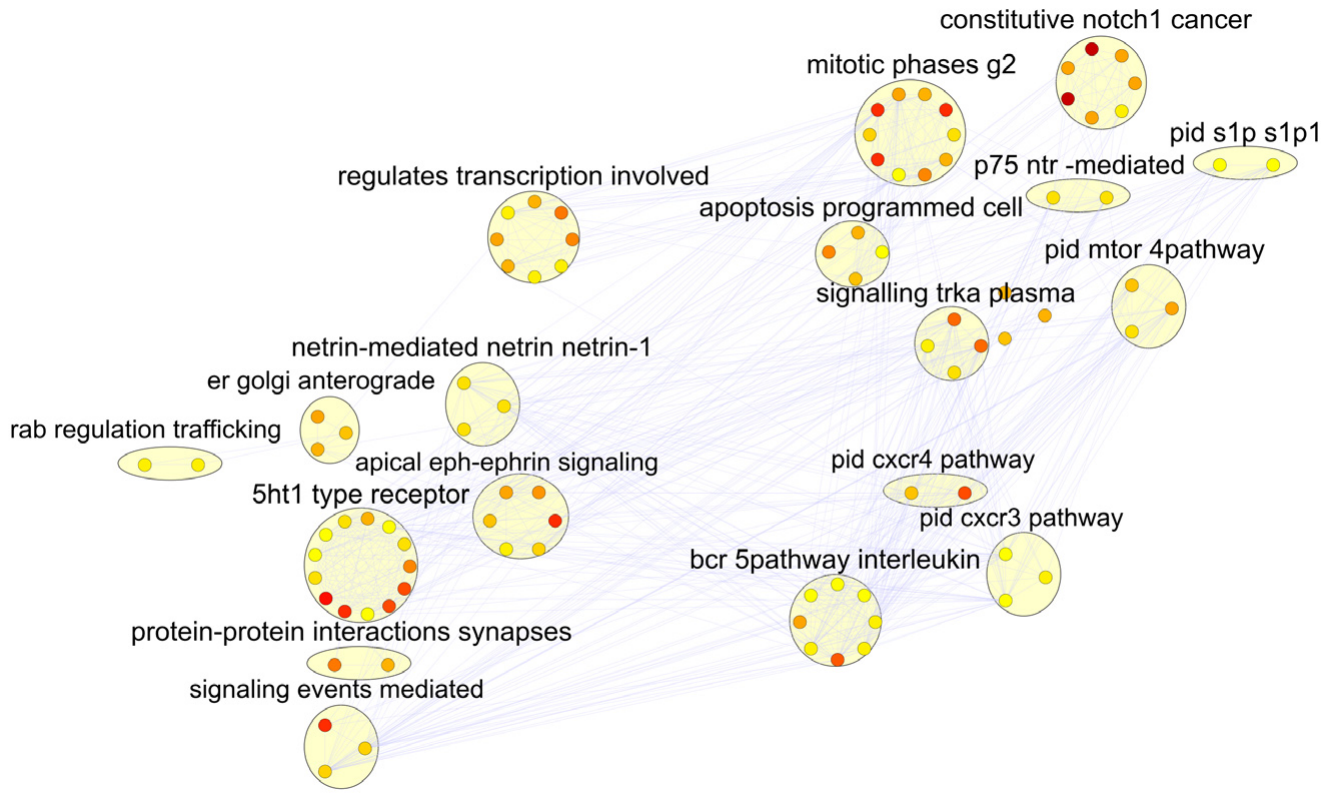
Enrichment map showing top-scoring pathway features for classifying case-control autism using rare CNVs. Nodes show pathway features cumulatively scoring 13 or higher out of 30, while edges connect pathways with common member genes. Node fill indicates pathway score, with yellow for the lowest and red for the highest.

The nodes in Figure 11 have been reorganized to group clusters sharing a broader theme. Terms related to neurotransmission and synaptic plasticity are in the bottom left, those related to the cell cycle and proliferation are in the top-right, and those related to immune function are in the bottom right.

**Figure 12.**
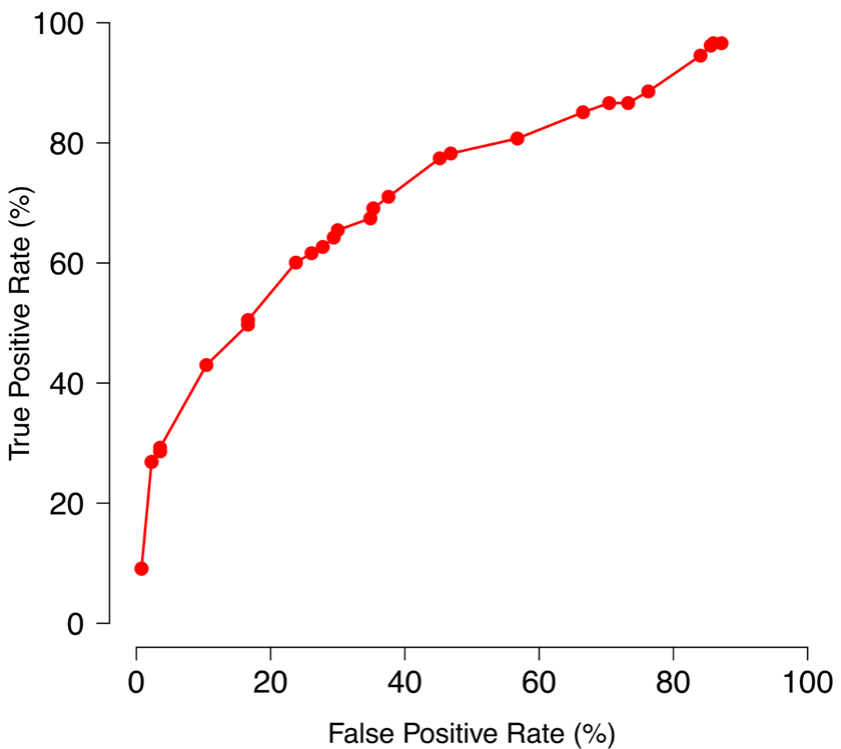
Model performance for case-control classification of autism using rare CNVs, with greater number of splits. Legend identical to Figure 9, except that here the graph is comprised of 30 measures because features are scored out of 30, rather than in Figure 9, where features are scored out of 9.

The dynamic range of feature scores is much larger as well, here ranging from 0 to 30. The resulting ROC curve in Figure 12 accordingly has 30 cutoffs at which specificity and sensitivity are evaluated, evidenced by 30 datapoints in that curve. This is in contrast to 9 cutoffs in the ROC curve shown in Figure 10.

### Use case 4: Mutation-based classifier using indirect mutations inferred from known interaction networks

netDx provides the option of reducing the sparsity of mutation data by inferring "indirect mutations" using prior knowledge of gene-gene interaction networks. Conceptually, the logic is that if a patient has a mutation in a given gene, the mutation indirectly impacts interacting genes. Indirect mutation is inferred by label propagating patient mutations over a gene-gene interaction network onto neighbours. The resulting smoothed network is then used for downstream applications. This network-based smoothing improved mutation-based tumour class discovery in four types of cancer ^12^. For label propagation, we use an R-based implementation of random walk with restart, a popular strategy in bioinformatic applications ^12– 15^. The result of using this strategy on a patient’s binary somatic mutation profile is a non-sparse profile in which genes are assigned a continuous score between zero and one, that reflects its network proximity to patient mutations. This propagation value is then ranked and binarized, with the top-ranked fraction set to one; this fraction defaults to 3% and is tunable. The binarization serves to limit inferred mutation to genes closest to the known mutations. For instance, genes distant from the patient's mutation would get a low propagation value, and would be thresholded to zero, i.e. not considered to be mutated. The result of this step is a less sparse binary matrix, which serves as input data to the predictor.

In this example, we use direct and inferred somatic mutations to classify Testicular Germ Cell Tumours (TGCT) ^16^ by binarized pathologic stage. As with the previous use case, we create pathway-level features to reflect that cancer progression occurs by a combination of genes acting in molecular networks corresponding to cancer hallmark processes such as cell proliferation and apoptosis ^13, 17^. As in Use Case 3, similarity used is the binary function. If two patients share a mutation in a pathway, their similarity for that pathway is one; otherwise it is zero.

***Setup***


set.seed(8)
suppressWarnings(suppressMessages(require(netDx)))
suppressWarnings(suppressMessages(require(MultiAssayExperiment)))


***Data.*** Clinical and genetic data are downloaded using the Bioconductor package *curatedTCGAData*. Mutations are converted to a binary matrix format where rows represent genes, columns represent patients; entry [i,j] is set to one if gene i has a somatic mutation, and zero otherwise.



genoFile <- paste(system.file("extdata",package="netDx"),
         "TGCT_mutSmooth_geno.txt",sep=getFileSep())
geno <- read.delim(genoFile,sep="\t",header=TRUE,as.is=TRUE)


phenoFile <- paste(system.file("extdata",package="netDx"),
         "TGCT_mutSmooth_pheno.txt",sep=getFileSep())
                                          
pheno <- read.delim(phenoFile,sep="\t",header=TRUE,as.is=TRUE)
rownames(pheno) <- pheno$ID

table(pheno$STATUS)

## 
## EARLY  LATE 
##    66    14


***Smooth mutations over a gene interaction network.*** The gene-gene interaction network used in this example contains high-confidence cancer-specific interactions ^18^. This specific network effectively clusters tumour samples of patients, distinguishing them by tumour type and time of survival. This is a binary symmetric network.


# download example nets from remote location for vignette
require(BiocFileCache)

## Loading required package: BiocFileCache

## Loading required package: dbplyr

netFileURL <- paste("http://download.baderlab.org/netDx/",
    "supporting_data/CancerNets.txt",sep="")
cache <- rappdirs::user_cache_dir(appname = "netDx")
bfc <- BiocFileCache::BiocFileCache(cache,ask=FALSE)
netFile <- bfcrpath(bfc,netFileURL)
cancerNets <- read.delim(netFile,sep="\t",header=TRUE,as.is=TRUE)
head(cancerNets[,1:5])

##        HSPA2 RPN1 GK2 HSPA6 PPP3R1
## HSPA2      0    1   1     1      1
## RPN1       1    0   0     1      0
## GK2        1    0   0     1      0
## HSPA6      1    1   1     0      1
## PPP3R1     1    0   0     1      0
## DLG1       1    0   0     1      0


*smoothMutations_LabelProp()* is used to smooth the mutations using the provided interaction network, by using label propagation. The output of this method is a continuous-valued network which reflects the network proximity of the non-zero values to the original mutations.


require(doParallel)

## Loading required package: doParallel

## Loading required package: foreach

## Loading required package: iterators

# Start the node clusters for parallel propagation
smoothedMutations <- smoothMutations_LabelProp(geno,cancerNets,numCores=1L)


Finally, the smoothed matrix is binarized. Genes with a propagation value greater than a specified cutoff are set to one, with the rest set to zero. This step ensures that genes which get a low propagation value are not used. Genes with lower smoothed values reflect those farther from the original mutation, and setting these to zero signifies a lack of confidence that these were impacted.


lessSparseMut <- thresholdSmoothedMutations(
   smoothedMutations,geno,"TGCT_CancerNets",c(20)
   )



***Create pathway-level features with binary patient similarity.*** Smoothed mutations are now grouped at the level of biological pathways. As with other examples, pathways are downloaded from a compilation of curated pathway databases (GMT format). Thereafter, we define pathway-level patient similarity to be binary; i.e. if two patients share a mutation in genes from the same pathway, their mutual similarity is one; else it is zero. Individual steps below use identical functions to those used in the first use case above.


#Setup to build the predictor
pathwayList <- readPathways(
   fetchPathwayDefinitions("January",2018)
   )
   
## ---------------------------------------

## Fetching http://download.baderlab.org/EM_Genesets/January_01_2018/Human/symbol/Human_AllPathways_January_01_2018_symbol.gmt

## File: 1c25416f319_Human_AllPathways_January_01_2018_symbol.gmt

## Read 3028 pathways in total, internal list has 3009 entries

##  FILTER: sets with num genes in [10, 200]

##    => 971 pathways excluded

##    => 2038 left

exprdat <- SummarizedExperiment(lessSparseMut, colData=pheno)
objList <- list(genetic=exprdat)


Now we define functions for patient similarity:


makeNets <- function(dataList,groupList,netDir,numCores,...) {
  netList <- c(); netList2 <- c()
  
  # create genetic nets
  if (!is.null(groupList[["genetic"]])) {
    netList <- makeMutNets(dataList[["genetic"]],
        groupList[["genetic"]],
        netDir,numC=numCores)
  } 
  return(netList)
}

# g geno matrix, genes by patients (columns) - binary
# pList list of genesets
# outDir - dir where nets are to be written
makeMutNets <- function(g,pList,oDir,numC) {
  g <- t(g) # transpose to have genes as columns
  cl    <- makeCluster(numC)
  registerDoParallel(cl)
  
  numPat <- c()
  netList <- foreach(k=1:length(pList)) %do% {
    idx <- which(colnames(g) %in% pList[[k]])
    
    if (length(idx)>0) {
      has_mut <- rowSums(g[,idx,drop=FALSE])
      has_mutp <- names(has_mut)[which(has_mut>0)]
      
      if (length(has_mutp)>=6) {
        ##cat(sprintf("%s: %i patients\n", names(pList)[k],
        ##            length(has_mutp)))
        #numPat <- c(numPat, length(has_mutp))
        pat_pairs <- t(combinat::combn(has_mutp,2));
        pat_pairs <- cbind(pat_pairs,1);
        outFile <- sprintf("%s/%s_cont.txt",oDir,names(pList)[k])
        write.table(pat_pairs, file=outFile,sep="\t",
                      col=FALSE,row=FALSE,quote=FALSE)
        basename(outFile)
      } else NULL
    } else {
      NULL
    }
  }
  stopCluster(cl)
  unlist(netList)
}


***Build predictor.*** Finally, we compile all the data into a MultiAssayExperiment object and as before, run the predictor.


exprdat <- SummarizedExperiment(lessSparseMut, colData=pheno)
objList <- list(genetic=exprdat)
groupList <- list(genetic=pathwayList)
dataList <- MultiAssayExperiment(objList,pheno)


The predictor call is essentially the same as with other simpler designs:


outDir <- paste(tempdir(),randAlphanumString(),"pred_output",sep=getFileSep())
if (!file.exists(outDir)) unlink(outDir,recursive=TRUE) 

out <- suppressMessages(
    buildPredictor(dataList=dataList,groupList=groupList,
      makeNetFunc=makeNets, ## custom similarity
      outDir=outDir, ## absolute path
      numCores=1L, featScoreMax=2L, featSelCutoff=2L,
      numSplits=2L,logging="none"
))



***Examine output.*** This code collects different components of model output to examine the results.


numSplits <- 2L
st <- unique(colData(dataList)$STATUS)
acc <- c()          # accuracy
predList <- list() # prediction tables

featScores <- list() # feature scores per class
for (cur in unique(st)) featScores[[cur]] <- list()

for (k in 1:numSplits) {
    pred <- out[[sprintf("Split%i",k)]][["predictions"]];
    # predictions table
    tmp <- pred[,c("ID","STATUS","TT_STATUS","PRED_CLASS",
                       sprintf("%s_SCORE",st))]
    predList[[k]] <- tmp 
    # accuracy
    acc <- c(acc, sum(tmp$PRED==tmp$STATUS)/nrow(tmp))
    # feature scores
    for (cur in unique(st)) {
       tmp <- out[[sprintf("Split%i",k)]][["featureScores"]][[cur]]
       colnames(tmp) <- c("PATHWAY_NAME","SCORE")
       featScores[[cur]][[sprintf("Split%i",k)]] <- tmp
    }
}


**Figure 13.**
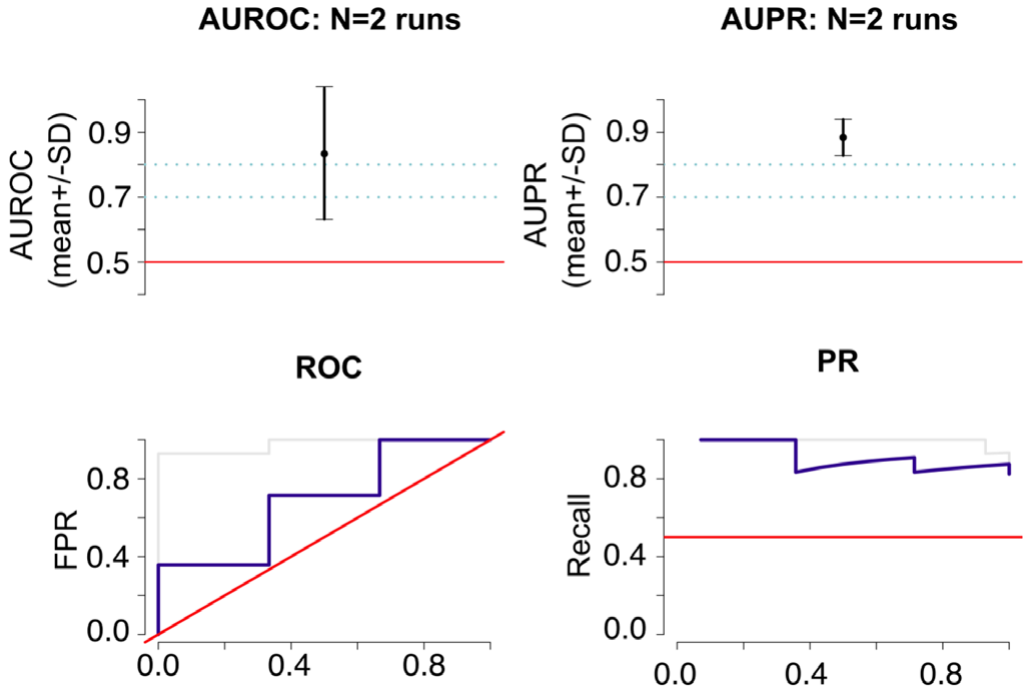
Performance measures for predictor that desparsifies binary somatic mutations using a user-provided gene-gene interaction network.


Plot the AUROC and AUPR curves ( Figure 13):

predPerf <- plotPerf(predList, predClasses=st)


Examine features with the highest scores. Here, these are pathways with somatic mutations that best predict vital status:


featScores2 <- lapply(featScores, getNetConsensus)
summary(featScores2)

##       Length Class      Mode
## EARLY 3      data.frame list
## LATE  3      data.frame list

featSelNet <- lapply(featScores2, function(x) {
     callFeatSel(x, fsCutoff=1, fsPctPass=0)
})
print(head(featScores2[["LATE"]]))

##                                                                                               PATHWAY_NAME
## 1                             1D-_I_MYO__I_-INOSITOL_HEXAKISPHOSPHATE_BIOSYNTHESIS_II__MAMMALIAN__cont.txt
## 2                                                                 3-PHOSPHOINOSITIDE_BIOSYNTHESIS_cont.txt
## 3                                                                  3-PHOSPHOINOSITIDE_DEGRADATION_cont.txt
## 4                                   ABORTIVE_ELONGATION_OF_HIV-1_TRANSCRIPT_IN_THE_ABSENCE_OF_TAT_cont.txt
## 5 ACTIVATED_PKN1_STIMULATES_TRANSCRIPTION_OF_AR__ANDROGEN_RECEPTOR__REGULATED_GENES_KLK2_AND_KLK3_cont.txt
## 6                                                     ACTIVATED_TAK1_MEDIATES_P38_MAPK_ACTIVATION_cont.txt
##   Split1 Split2
## 1      1     NA
## 2      2      2
## 3      2      2
## 4      1     NA
## 5      2      2
## 6      2      1


## Software updates

netDx v1.1.4 has several updates relative to the version released with the netDx methods report (v1.0.23) ^2^. The new netDx package supports OS X and Unix platforms. It also supports Windows systems, with the exception of those that do not have the Java executable available in the system search path. The companion R package *netDx-examples*, previously used to store example data, is now deprecated. All examples are now either contained within the *netDx* package or are fetched from Bioconductor using local file-caching via the *FileCache* package. Major functions have been renamed to reflect their role rather than implementation, making their usage more intuitive ( Table 1). The current version of netDx includes a novel workflow of building a classifier from sparse genetic data (see *Use Case 3*), using the function *buildPredictor_sparseGenetic().* We also added the functionality to generate an integrated patient similarity network from features passing selection. The *plotIntegratedPatientNetwork()* function generates this network, computes statistics on pairwise shortest distance measures (Dijkstra distance) within and across labels, and automatically generates a network visualization in Cytoscape.

A number of software updates were made as part of Bioconductor integration. Unlike the previous version where all user output was written to a specific output directory, all predictor output is now returned to users as R objects, and intermediate work is written to temporary directories by default. The turnkey predictor-building function no longer automatically generates a log file; rather, users are required to create their own log files using the R *sink()* function. Functions computing model performance and plotting no longer assume a directory structure created by the model-building step. Users now set random number generator seeds at the outset, instead of providing a seed as an input parameter to various functions. Automated network visualization in Cytoscape now uses *RCy3*, for programmatic access of Cytosape from R.

Memory improvements were made to the underlying GeneMANIA network integration algorithm Java implementation ^19, 20^, creating a modified version specifically for netDx. netDx incurs a relatively higher memory footprint because each feature in netDx internally generates a similarity network with pairwise similarity measures. Network integration, a step in feature selection, requires keeping all these networks in memory. Certain grouping rules also incur a greater memory footprint than others. Notably, a model with pathway-level features converts one gene expression data matrix into ~2,000 pathway-level patient similarity networks; such a design is less scalable in the number of nodes, than one which creates a single feature based on all gene expression. We optimized netDx memory usage by customizing the underlying GeneMANIA Java application used for network integration. netDx uses a modified version of the GeneMANIA implementation, which bypasses steps not required for the netDx pipeline, such as the identifier conversion and steps involving file input/output. Memory and computational time improvements were benchmarked by building binary classifiers for breast tumours and schizophrenia case-control classification. The CommonMind Consortium ^21^ dataset (downloaded from Synapse: syn5607607) included 279 controls and 258 cases, with a total of 537 patients, with gene expression data from the prefrontal cortex organized into pathway level features (1,735 pathways). The breast cancer data was part of the TCGA project ^9^, with tumour gene-expression for 348 patients, including 154 Luminal A and 194 tumours of other subtypes, also organized into pathway-level features (1,706 pathways). In the benchmark, an approximately 70:30 split of samples was used for cross validation. We measured training time for the predictor using the 70% of samples of a single subtype. All tests were performed on an Intel Xeon @ 2.6GHz machine with 126 GB of available RAM and 12 cores. During benchmarking, threads had a fixed amount of RAM available, with discrete steps of 4 GB, 6 GB and 8 GB. Here each predictor was built using only a single core. Benchmarking runs were parallelized using GNU parallel ^22^, where the performance was averaged over four runs of the 10 queries for each datasets. Following improvements, memory use dropped to one-third of the original amount. With the updated software, the CommonMind dataset also required two-thirds of the time to build the predictor, as compared to with the original version ( Table 2).

**Table 2.** Benchmarking performance improvement for netDx. Computation times are averaged over four runs of the same ten queries for feature-scoring one patient label, while limiting the executable to a single core. All tests were performed on an Intel Xeon @ 2.6GHz machine with 126GB of available RAM and 12 cores.

JavaMemory setting	Previous runtime (s) v0.99	Current runtime (s) with percent improvement v1.4
Breast cancer (Luminal A): 111 patients, 1706 pathway-based networks from gene expression data		
4GB	273.95 +/- 13.97	167.73 +/- 5.21 (38%)
6GB	275.11 +/- 12.35	166.89 +/- 4.81 (39%)
8GB	273.74 +/- 13.44	167.24 +/- 4.44 (39%)
Schizophrenia (case): 185 patients, 1735 pathway-based networks from gene expression data		
4GB	552.41 +/- 26.06	389.37 +/- 11.26
6GB	549.31 +/- 23.83	388.22 +/- 9.14
8GB	547.47 +/- 21.93	391.85 +/- 10.93

Finally, the feature selection step now provides the option of using a Monte Carlo resampling strategy for selecting samples for iterative feature scoring. The previous version of the software required a fraction of samples to be held out, the fraction being directly related to the maximum feature score. The Monte Carlo resampling approach should allow users to increase the upper-bound of feature scores, even in smaller samples.

## Conclusions

The updated netDx software provides an improved user experience for clinical research applications pertaining to risk stratification, treatment response, and to identify biomarkers associated with patient subtypes. Method extensions will be required for further feature additions, such as the ability to predict continuous outcome. Classification of borderline patients could be better controlled, perhaps by a user-specified margin. Similar to pathway-level grouping for gene expression data, other grouping strategies will be required for other types of genomic data, such as miRNA, single nucleotide polymorphisms, and brain imaging data.

## Data availability

### Underlying data

Data for the autism case/control classification ^5^ is provided as part of the netDx package.

Data for the breast cancer example is from The Cancer Genome Atlas ^9, 23^. They are fetched from the *curatedTCGAData* package which is maintained by the Bioconductor repository.

netDx vignettes are available at: https://bioconductor.org/packages/release/bioc/html/netDx.html

## Software availability

netDx is available from Bioconductor: http://bioconductor.org/packages/devel/bioc/html/netDx.html

Source code available from: https://github.com/BaderLab/netDx.

Archived source code at time of publication: http://doi.org/10.5281/zenodo.4048852 ^3^.

Issue tracker: https://github.com/BaderLab/netDx/issues

License: MIT License
