# Supplementary material for: netDx: Software for building interpretable patient classifiers by multi-'omic data integration using patient similarity networks
Source: F1000Res. 2021 Jan 22;9:1239. Originally published 2020 Oct 15. [Version 2] doi: 10.12688/f1000research.26429.2 (PMC7883323; doi:10.12688/f1000research.26429.2)
Supplement: Peer review history

# Peer review: Reviewer response for version 2

**DOI:** 10.5256/f1000research.33819.r77914

**Published:** 2021-02-12

**Authors:** Kim-Anh Lê Cao

**Affiliations:** 1 University of Melbourne, Melbourne, Australia

I am happy with these revisions, well done!

Are the conclusions about the tool and its performance adequately supported by the findings presented in the article?

Yes

Is the rationale for developing the new software tool clearly explained?

Yes

Is the description of the software tool technically sound?

Yes

Are sufficient details of the code, methods and analysis (if applicable) provided to allow replication of the software development and its use by others?

Partly

Is sufficient information provided to allow interpretation of the expected output datasets and any results generated using the tool?

Yes

Reviewer Expertise:

Computational statistics, multi omics integration, R software development.

I confirm that I have read this submission and believe that I have an appropriate level of expertise to confirm that it is of an acceptable scientific standard.

# Peer review: Reviewer response for version 2

**DOI:** 10.5256/f1000research.33819.r77913

**Published:** 2021-02-09

**Authors:** Anais Baudot, Judith Lambert, Laurent Tichit

**Affiliations:** 1 INSERM, MMG, Aix Marseille University, Marseille, France; 2 I2M, Institut de Mathématiques,, Aix Marseille University, Marseille, France

We still obtained some errors while running the code with Linux/Windows/French locale. However, all four use cases were running correctly using the docker image of the working environment.

Are the conclusions about the tool and its performance adequately supported by the findings presented in the article?

Yes

Is the rationale for developing the new software tool clearly explained?

Yes

Is the description of the software tool technically sound?

Yes

Are sufficient details of the code, methods and analysis (if applicable) provided to allow replication of the software development and its use by others?

Partly

Is sufficient information provided to allow interpretation of the expected output datasets and any results generated using the tool?

Yes

Reviewer Expertise:

Systems and Network Biology, Bioinformatics, Computational Biology.

We confirm that we have read this submission and believe that we have an appropriate level of expertise to confirm that it is of an acceptable scientific standard.

# Peer review: Reviewer response for version 1

**DOI:** 10.5256/f1000research.29179.r73050

**Published:** 2020-11-25

**Authors:** Anais Baudot, Judith Lambert, Laurent Tichit

**Affiliations:** 1 INSERM, MMG, Aix Marseille University, Marseille, France; 2 I2M, Institut de Mathématiques,, Aix Marseille University, Marseille, France

The paper entitled “netDx: Software for building interpretable patient classifiers by multi-'omic data integration using patient similarity networks” is a companion paper to the article “netDx: interpretable patient classification using integrated patient similarity networks“ published in Plos Comp Bio in 2019. Its goal is to present an updated version of the R software implementation of netDx as a Bioconductor package.

 The netDx tool proposes an approach to building a patient classifier from heterogeneous patient data, from clinical to omics. The availability as an R package is of interest to the community. In addition, the manuscript details 4 different uses cases that could help interest readers to apply the tools. We however had difficulties running the code provided in the use cases and obtained different errors and warnings, so have been in contact with the authors to try to solve the problems. However, debugging code necessitate a lot of exchanges, and hundreds of lines of error outputs cannot go in a peer review. These problems are not solved yet. 

****

Are the conclusions about the tool and its performance adequately supported by the findings presented in the article?

Yes

Is the rationale for developing the new software tool clearly explained?

Yes

Is the description of the software tool technically sound?

Yes

Are sufficient details of the code, methods and analysis (if applicable) provided to allow replication of the software development and its use by others?

Partly

Is sufficient information provided to allow interpretation of the expected output datasets and any results generated using the tool?

Yes

Reviewer Expertise:

Systems and Network Biology, Bioinformatics, Computational Biology

We confirm that we have read this submission and believe that we have an appropriate level of expertise to confirm that it is of an acceptable scientific standard, however we have significant reservations, as outlined above.

# Response

**Published:** 2020-12-22

**Authors:** Shraddha Pai

**Affiliations:** University of Toronto, Canada

Responses are shown in bold beneath each reviewer comment, the latter shown in italics.

 ---

*The paper entitled “netDx: Software for building interpretable patient classifiers by multi-'omic data integration using patient similarity networks” is a companion paper to the article “netDx: interpretable patient classification using integrated patient similarity networks“ published in Plos Comp Bio in 2019. Its goal is to present an updated version of the R software implementation of netDx as a Bioconductor package.*

**

*The netDx tool proposes an approach to building a patient classifier from heterogeneous patient data, from clinical to omics. The availability as an R package is of interest to the community. In addition, the manuscript details 4 different uses cases that could help interest readers to apply the tools. We however had difficulties running the code provided in the use cases and obtained different errors and warnings, so have been in contact with the authors to try to solve the problems. However, debugging code necessitate a lot of exchanges, and hundreds of lines of error outputs cannot go in a peer review. These problems are not solved yet.*

**Response: We thank the reviewers for taking the time and effort to work with us to resolve these issues. There were three main sources of errors that the reviewers encountered. The resolution for each is described below.**

****

**To better support Windows users we now provide Docker images of working environments with the latest version of netDx. The following text has been added to the “Operation” section: “Windows users can access netDx via a Docker image provided at https://hub.docker.com/repository/docker/shraddhapai/netdx.”**

1. **Incompatibility with French locale: The reviewers tested netDx on a system with a French locale, which identified an unforeseen international incompatibility. netDx uses a Java-based network integration software during feature selection. Parts of this software would break when provided with numbers using a comma for a decimal separator; as such they were incompatible with several non-English locales. We have now fixed the issue in netDx v1.3.1 to ensure that all files passed from R to Java are forced to use a period as decimal separator.**
****
** We are now able to successfully run all vignettes in a French locale; please see https://hub.docker.com/repository/docker/shraddhapai/netdx/general (Tag: v1.3.1_french). Note that French locale users do not have to download this specific version of netDx; the Docker image is provided as a contained environment with a French locale, where we have demonstrated that netDx now works.**
2. **Windows incompatibility: netDx is currently not supported on some versions of the Windows operating system because of variation in how Java is invoked by different Java versions.  In particular, newer Windows systems do not have a java executable available on the search path by default. We have noted this in the current version of the manuscript.**
****
** Therefore for now we will continue to support netDx on OS X and Unix systems in BioConductor, and will provide a Docker container for Windows users.**
****
** A working Docker image, supporting Windows and other operating systems that support Docker, is available on Docker hub:**
**https://hub.docker.com/repository/docker/shraddhapai/netdx/general (Tag: v1.3.1)**
3. **Use case 4 had outdated function calls. That has been amended in the current version of the manuscript. We also supplied the reviewers with the updated vignette when we learnt about the error.**
****
** We hope that the resolution of the above three issues is satisfactory. We are working with the reviewers to ensure that they are able to run the software given the changes above.**

# Peer review: Reviewer response for version 1

**DOI:** 10.5256/f1000research.29179.r73048

**Published:** 2020-10-29

**Authors:** Kim-Anh Lê Cao

**Affiliations:** 1 University of Melbourne, Melbourne, Australia

The authors illustrate full R workflows to apply netDx using four case studies with various ranges of classification difficulty and data analysis settings. The netDx package proposes various ways of grouping features, for example using known biological pathways, and several graphical outputs. I appreciated that netDx builds on MultiAssayExperiment for easier handling of multi omics data sets. The manuscript will be useful for readers eager to get started with netDx.

 Below are some suggestions for improvement of the manuscript.

 Methodological aspects:

- I acknowledge that the original algorithm has been detailed in reference [2], however, the present manuscript gives some emphasis on the ability of netDx to handle missing values. A short statement describing how this is done would be helpful.
- I suggest rewriting the sentence 'The final model is created by choosing features that consistently score highly.' in the introduction. On a first read, it appeared as if there was selection bias during the process.
- I have some reservations regarding the representation of SEM in the AUROC figures, why not using SD?

Implementation aspects:

- While much effort and improvements have been done in the netDx package v1.1.4, I believe that additional functions could be created to be user friendly. For example, many customs functions (e.g. makeNets, the code proposed to reformat the results and calculate the accuracy or to extract the results to Cytoscape, to list a few) could be recoded into more generic functions. I would encourage the authors to revisit the code they propose in these workflows and improve when possible. 
- I am not sure Table 1 column 3 (function name in v1.0.23) and part of the software update paragraph is useful here. Presumably this could appear on the GitHub page and the NEWS files, unless the objective of this manuscript is also to update the users of the latest changes.

Minor typos:

- 'published' in the Introduction.

Are the conclusions about the tool and its performance adequately supported by the findings presented in the article?

Yes

Is the rationale for developing the new software tool clearly explained?

Yes

Is the description of the software tool technically sound?

Yes

Are sufficient details of the code, methods and analysis (if applicable) provided to allow replication of the software development and its use by others?

Partly

Is sufficient information provided to allow interpretation of the expected output datasets and any results generated using the tool?

Yes

Reviewer Expertise:

Computational statistics, multi omics integration, R software development.

I confirm that I have read this submission and believe that I have an appropriate level of expertise to confirm that it is of an acceptable scientific standard, however I have significant reservations, as outlined above.

# Response

**Published:** 2020-12-22

**Authors:** Shraddha Pai

**Affiliations:** University of Toronto, Canada

Responses are shown in bold under corresponding reviewer comments shown in italics.

 ---

*The authors illustrate full R workflows to apply netDx using four case studies with various ranges of classification difficulty and data analysis settings. The netDx package proposes various ways of grouping features, for example using known biological pathways, and several graphical outputs. I appreciated that netDx builds on MultiAssayExperiment for easier handling of multi omics data sets. The manuscript will be useful for readers eager to get started with netDx.*

**We thank the reviewer for their time, appreciative comments, and feedback. We also hope the manuscript helps new users get started with netDx for classification and data integration.**

*Below are some suggestions for improvement of the manuscript.*

*Methodological aspects:*

- *I acknowledge that the original algorithm has been detailed in reference [2], however, the present manuscript gives some emphasis on the ability of netDx to handle missing values. A short statement describing how this is done would be helpful.*

**Response: Text added in introduction of use case 3:**

**“netDx handles missing data at two levels. First, netDx uses patient similarity networks, not input data, as its features. Missing data can be handled by the similarity metric used to make this conversion. e.g. If similarity is defined as the Pearson correlation between gene expression measures at the pathway level, then omitting missing genes from the correlation calculation still allows the correlations, and thus the pathway-level network,, to be computed. Where patients are missing a particular feature, the network integration step uses what information it has. For example, in a scenario where the data consist of transcriptomic and proteomic measures, if a patient is missing transcriptomic data, the integration step will use only the proteomic data (edges) for that patient (network edges) for that patient.”**

- *I suggest rewriting the sentence 'The final model is created by choosing features that consistently score highly.' in the introduction. On a first read, it appeared as if there was selection bias during the process. *

**Response: We altered the sentence in the manuscript to: “The final model is created from features that scored highly in feature selection, a step that uses only training samples”. **

- *I have some reservations regarding the representation of SEM in the AUROC figures, why not using SD?*

**Response: We have now changed the function that plots the AUROC curve to use standard deviation as default, and have provided the user the option of using SEM. The corresponding figures have been updated in the manuscript to show the change (Figure 3 and Figure 13).**

*Implementation aspects:*

- *While much effort and improvements have been done in the netDx package v1.1.4, I believe that additional functions could be created to be user friendly. For example, many customs functions (e.g. makeNets, the code proposed to reformat the results and calculate the accuracy or to extract the results to Cytoscape, to list a few) could be recoded into more generic functions. I would encourage the authors to revisit the code they propose in these workflows and improve when possible.  *

**Response: We agree and will continue to create useful utility functions to make it easier for new users to use in future releases of netDx. In order to avoid potentially over complicating or overengineering our API, we are waiting for user feedback before making these additions.**

- *I am not sure Table 1 column 3 (function name in v1.0.23) and part of the software update paragraph is useful here. Presumably this could appear on the GitHub page and the NEWS files, unless the objective of this manuscript is also to update the users of the latest changes.*

**Response: We agree and have removed this column from Table 1.**

*Minor typos:*

- *'published' in the Introduction.*

**Response: Corrected.**
